# Promising alternatives of CD47 monoclonal antibody: an injectable degradable hydrogel loaded with PQ912 for postoperative immunotherapy effectively blocks CD47-SIRPα signal

**DOI:** 10.7150/thno.72310

**Published:** 2022-05-27

**Authors:** Chang Li, Yubo Liu, Dan Li, Qiu Wang, Shuang Zhou, Haotian Zhang, Yongjun Wang, Zhonggui He, Hongzhuo Liu, Jin Sun

**Affiliations:** 1 Wuya College of Innovation, Shenyang Pharmaceutical University, Shenyang, 110016, P. R. China.; 2 School of Life Science and Biopharmaceutics, Shenyang Pharmaceutical University, Shenyang, 110016, P. R. China.

**Keywords:** PQ912, CD47-SIRPα, cancer immunotherapy, hydrogel scaffold, red blood cells

## Abstract

**Rationale**: Many cancers have evolved different mechanisms to evade immune surveillance. Macrophages, the innate defense of the immune system, are limited in their phagocytosis by CD47 anti-phagocytic signaling expressed on the surface of tumor cells. Although the CD47 monoclonal antibody (aCD47) strategy has been extensively studied in clinical trials, the depletion of aCD47 by red blood cells (RBCs) and the resulting hematotoxicity have impeded their application in tumor treatment.

**Methods:** Here, we reported an injectable hydrogel scaffold that allowed for local delivery of small-molecule inhibitor PQ912. The biodegradable hydrogel scaffold (PQ/PB-Gel) was formed by rapid cross-linking of tetra-armed PEG succinimidyl succinate (Tetra-PEG-SS) solution and alkalescent bovine serum albumin (BSA) solution through ammonolysis reaction.

**Results:** PQ/PB-Gel had excellent effect on inhibiting local recurrence of two kinds of tumors. The hydrogel system inhibited the generation of “don't eat me” signals during the treatment cycle by inhibiting the expression of newly generated neoplastic CD47. Thus, it avoided adverse reactions such as erythrocytopenia after the use of aCD47 in terms of safety. After the “don't eat me” signal was blocked the clearance and recognition of cancer cells by macrophages and antigen-presenting cells were enhanced, sequentially systemic immune response was activated and further memory T lymphocyte (T cell) formation was induced.

**Conclusions:** PQ/PB-Gel had a simple preparation and administration method, low production cost, excellent efficacy and low toxicity, so it had good practicability. This might provide a safe alternative strategy for aCD47 for inhibit local tumor recurrence and distal metastasis in postoperative immunotherapy.

## Introduction

Immune checkpoint inhibitors (ICIs) have remarkably changed the treatment modality of several advanced malignancies and revolutionized the landscape of cancer therapy. The existing ICIs targeting T-cell inhibitory receptors, such as the antibodies against PD-1, PD-L1, and CTLA4, can induce complete and durable antitumor immunity in patients with metastatic and refractory cancers 1. However, the response rate of these inhibitors in cancer patients is still unsatisfactory 2. Therefore, the importance of improving tumor antigen processing and innate immunity in tumor cell monitoring and eradication has been increasingly recognized.

Over the past decade, macrophages, a key effector of innate anti-tumor immunity, have been thrust into the spotlight. Promising evidence from preclinical studies has demonstrated that targeting macrophage phagocytosis is an effective treatment strategy, either alone or in combination with other therapies 3. However, its clinical application is still subject to some challenges related to efficacy and safety. (i) Efficacy: many cancers have evolved different mechanisms to evade immune surveillance. As the natural defense of the immune system, the phagocytosis of macrophages is limited by CD47 anti-phagocytosis signal expressed on the surface of tumor cells. In addition, the acidic environment formed by the high concentration of lactic acid produced by tumor metabolism not only increases the proportion of M2-like tumor-associated macrophages (M2-like TAMs) 4, 5, but also inhibits T cell function, protecting tumor cells from the killing of the immune system. (ii) Safety: blocking the interaction between CD47 and the signal-regulating protein α (SIRPα) expressed on phagocytes can promote phagocytosis of phagocytes against tumor cells. In addition, there are growing evidences that blocking the CD47-SIRPα axis also enhances the function of antigen-presenting cells, thereby stimulating T-cell-mediated antitumor immunity 5-10. Currently, aCD47 is one of the most widely studied therapeutic agents targeting CD47-SIRPα in clinical trials. However, the depletion of aCD47 by CD47 expressed on RBC and the resulting hematotoxicity (anemia, thrombocytopenia) hinder its application in tumor therapy 11-14.

These limitations have led to a focus on developing new approaches to extend its therapeutic potential to a wider range of patients in a safer and more controlled manner. Recently, small-molecule inhibitors that interrupt the CD47-SIRPα axis have become a research hotspot. Compared with monoclonal antibodies, small-molecule inhibitors have the following advantages: i) the small-molecule inhibitors undergo rapid elimination and flexible drug exposure *in vivo*, resulting in less side effects, ii) They have the ability to achieve deep drug penetration into tumors where antibodies are unable to access 15. Recent studies have demonstrated that glutaminyl cyclase isoenzyme (QPCTL) inhibitors regulating CD47 pyroglutamate formation possess high potency to interfere with CD47-SIRPα interaction 16. More importantly, the QPCTL inhibitors only interrupt the formation of CD47 but show less effect on mature CD47, indicating less adverse effects 17. Among all the investigated QPCTL inhibitors, only PQ912 entered the clinical trials. The current study investigated the efficacy of PQ912 in the treatment of early-stage Alzheimer's disease and completed a phase 2 clinical trial in 2017 (NCT02389413). A more recent study reported a stimuli-responsive photothermal nanoplatform combining PQ912 and aPD-1 analog to improve the antitumor immune effect in 4T1 model 18, with little effort devoted to exploiting PQ912 for therapeutic applications in preventing local and distant recurrence. Injectable hydrogels are local drug delivery strategies. Compared with systemic drug delivery methods, local drug delivery methods have the advantages of (i) higher dose of targeted site delivery; (ii) can reduce the toxic and side effects on other organs and tissues; (iii) easy control and sustained-release drug delivery.

**Figure 1:**
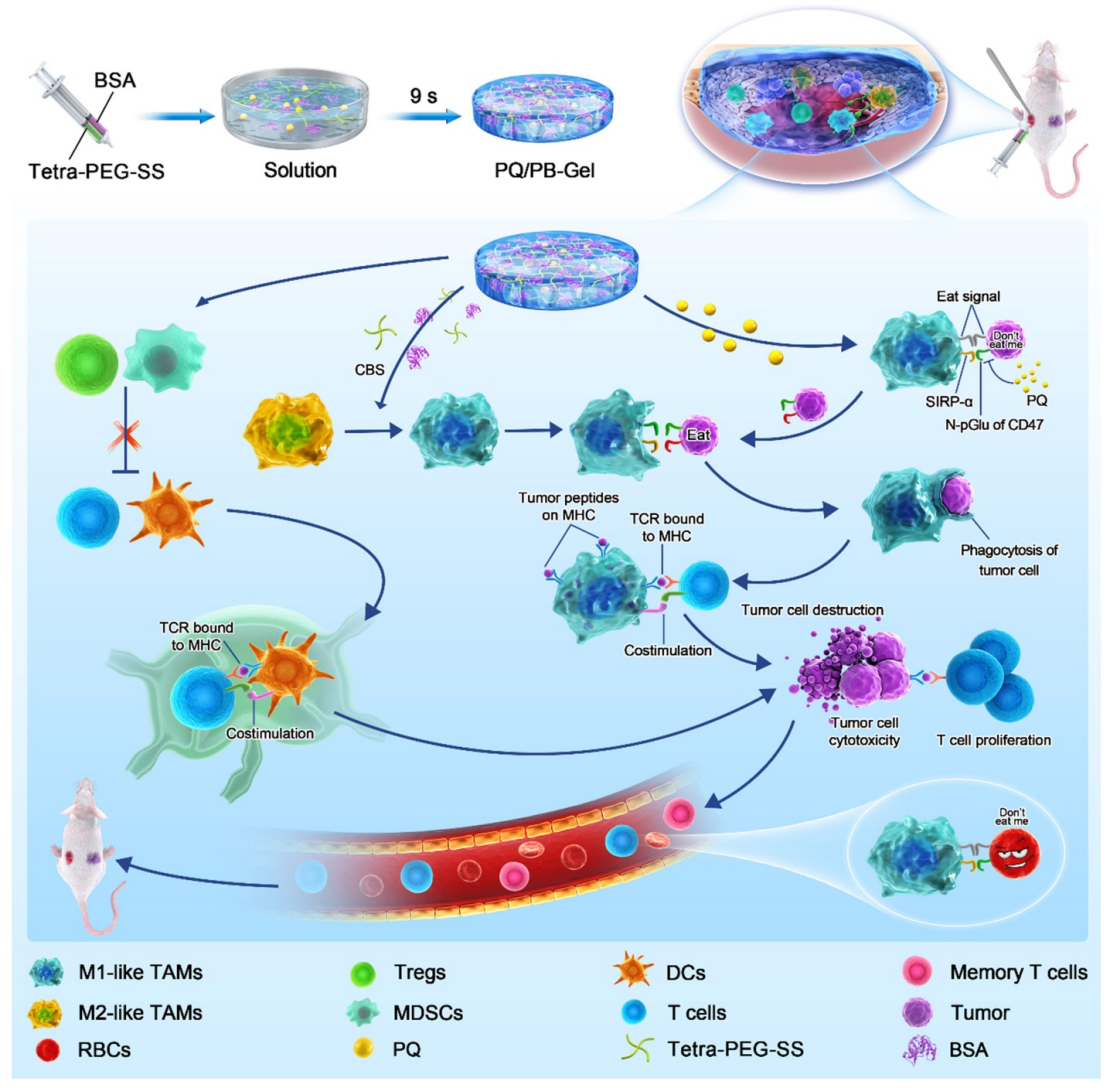
Schematic diagram of immunotherapeutic strategy based on small-molecule inhibitors TME remodeling hydrogel scaffolds. Tetra-PEG-SS and BSA were rapidly cross-linked under alkaline conditions through a mixer at the front of a two-component syringe to form a hydrogel. The weakly basic system of hydrogel matrix dissolved in carbonate buffer solution (CBS) promoted the activation of M1-like TAMs. The gel matrix showed some reversal of the temporary immunosuppression associated with wound healing caused by the resection of the primary tumor. *In situ* delivery of PQ912 via an injectable hydrogel interfered with CD47 pyroglutamate (pGlu) formation on the tumor cell surface and enhanced the function of antigen-presenting cells, thereby stimulating T cell-mediated antitumor immunity and memory T cell formation while protecting RBCs from damage.

Herein, we designed injectable gel containing therapeutic drugs that can be applied to the surgically resected wound site and hopefully achieve an immune response *in situ*, avoiding the complex process of cell engineering. Tetra-PEG-SS and BSA could form hydrogel rapidly via ammonolysis reaction under physiological conditions. It was worth noting that hydrogel scaffolds could not only act as sealants to reduce postoperative complications, but also acted as a repository to regulate the release of small molecule therapeutic drugs. *In situ* local administration of PQ912 via an injectable hydrogel down-regulated the expression of CD47 on the tumor surface and enhanced the function of antigen-presenting cells, thereby stimulating T cell-mediated antitumor immunity (Figure 1). Specially, *in situ* delivery of immune-stimulating compounds at the tumor-removed site could be used to exploit immunomodulatory intervention during surgery in the elimination of primary tumor-associated immunosuppressive effects and metastasis. This strategy with the rational combination of tumor surgery and immunotherapy provides a promising method for the inhibition of tumor recurrence and distal metastasis.

## Methods

### Materials and animals

PQ912, sulfonated butyl ether β-cyclodextrin derivatives (SBE-β-CD) were purchased from MCE (Shanghai, China). BSA and mouse lymphocyte separator kit were purchased from Beijing Solarbio Science & Technology Co., Ltd. (Beijing, China). Tetra-PEG-SS was obtained from Beijing JenKem Technology Co., Ltd. (Beijing, China). Na_2_CO_3_ and NaHCO_3_ were obtain from Shanghai Haohong Scientific Co., Ltd., Leyan. D-luciferin, potassium salt and 3-(4,5-Dimethylthiazol-2-yl)-2,5-diphenyl tetrazolium bromide (MTT) were obtained from Dalian Meilun Biotech Co., Ltd. (Dalian, China). Anti-CD45-Percp/Cy5.5 was purchased from Elabscience Biotechnology Co., Ltd. (cat. no. E-AS-F1136J). aCD47 and anti-PD-1 antibodies (aPD-1) and the other fluorescence coupled flow cytometry were purchased from Biolegend. Erythrocyte lysate was obtained from Beijing Biomed Gene Technology Co., Ltd.

Female C57BL/6 and BALB/c mice (6-8 weeks old) used in the experiments were supplied by the Animal Centre of Shenyang Pharmaceutical University (Shenyang, China). All the animal involved experiments were carried out according to the Guide for Care and Use of Laboratory Animals of Shenyang Pharmaceutical University.

### Formation and characterization of PQ/PB-Gel

PB-Gel was obtained by mixing weakly alkaline BSA solution (Component A) and neutral Tetra-PEG-SS solution (Component B) in equal volume through a two-component syringe. Component A was composed of BSA dissolved in weakly alkaline carbonate buffer solution (CBS) at a concentration of 10 mg/100 μL. Component B was the solution of Tetra-PEG-SS in PBS (7.4) with the concentration to be 10% (w/v). The formation of PQ/PB-Gel was same as PB-Gel except that PQ912 was pre-dissolved in a CBS containing SBE-β-CD.

In order to study the effect of the pH of weakly basic CBS on the formation time of hydrogel, BSA was dissolved in the CBS of pH 9, 10, 11, respectively. The remaining steps were as described above, and the time of hydrogel formation was recorded with a timer.

The morphologies of PB-Gel were characterized by using SEM after freeze-drying. Rheological measurements were performed using AR2000EX at 25 °C. We measured frequency sweep of PB-Gel. An angular frequency ω of 15 rad/s and a deformation amplitude γ of 0.5% was selected to perform the rheological studies.

### In vivo release and degradation of PB-Gel

To evaluate the release profile of small molecule compound *in vivo*, rhodamine B (Rom) was as a model compound for PQ912. PB-Gel containing Rom (Rom/PB-Gel) was *in situ* treated surgically into non-tumor-bearing mice. As a control, Rom/PB-Sol (a mixed solution of Rom, BSA and Tetra-PEG-SS) was injected into the incision site. Fluorescence intensity was monitored and analyzed at different time points using the* in vivo* imaging system (IVIS) spectrophotometric imaging system (Perkin Elmer). After PB-Gel was *in situ* treated surgically into mice, the degradation of PB-Gel *in vivo* was monitored by observing the size of Gel lump in abdominal bulge.

### Release of PQ912 in vitro

To determine the release kinetics of PQ912 from hydrogel, the PQ/PB-Gel was immersed in 1 mL PBS (pH 6.5) containing 0.5% SBE-β-CD at 37 ℃. At each time point, removed 200 μL of the medium and added the equal volume of fresh buffer. The content of the drug was determined by HPLC at 254 nm. The mobile phase was acetonitrile: water + 0.1% TFA = 80:20. The flow rate and injection volume were 1 mL/min and 10 μL, respectively.

### Cell culture

Cell-culture dishes were commercially available from NEST Biotechnology (Wuxi, China). Penicillin-streptomycin solution was purchased from Procell Life Science & Technology Co., Ltd. Stable luciferase expression mouse mammary carcinoma cell lines (4T1-luc) and RAW 264.7 macrophages were obtained from Peking Union Cell Bank (Beijing, China). Stable luciferase expression murine melanoma cells (B16F10-luc) were obtained from Shanghai MEIXUAN Biological Science and Technology Ltd. (Shanghai, China). 4T1 cells expressing red fluorescent protein (RFP-4T1) cells were purchased from Tongpai Biotechnology Co. Ltd (Shanghai, China). RAW 264.7 cells were cultured in DMEM medium containing streptomycin (100 µg/mL), 10% FBS, and penicillin (100 units/mL). 4T1-luc, B16F10-luc and RFP-4T1 cells were cultured in 1640 RPMI medium containing streptomycin (100 µg/mL), 10% FBS, and penicillin (100 units/mL). The culture conditions for the cells were 5% CO_2_ at 37 °C.

### Cytotoxicity evaluation of PQ912 in vitro

Both the B16F10 and 4T1 cell lines were used to evaluate the relative cytotoxicity of the PQ912 at different concentrations. The corresponding cells (2000 cells/well) were inoculated in 96-well plates and incubated with corresponding series of concentrations of PQ912 at 37 ℃ for 24, 48, and 72 h. At the end of incubation period, each well was treated with 20 μL MTT (5 mg/mL) for 4 h. The resulting formazan crystals were dissolved in DMSO, and the absorbance was recorded at 570 nm with Varioskan Flash multimode microreader (Thermo Scientific, USA). The experiments were repeated three times, using untreated cells as controls.

### In vitro phagocytosis assay

RAW 264.7 macrophages were prelabeled with carboxyfluorescein succinimidyl amino ester (CFSE) as per the manufacturers protocol. RFP-4T1 cells were treated with the groups include PBS, PQ912 (50 μM), aCD47 (5 μg/mL) and PQ912 + aCD47 for 24 h, respectively. The treated RFP-4T1 cells were co-cultured with CFSE stained RAW 264.7 macrophages at placed in 12-well plates at a 1:2 ratio. The culture medium was replaced with a serum-free mixture of 1640 RPMI and DMEM medium. After incubation for 4 h, the collected cells were washed with PBS for 3 times and resuspended with PBS containing 0.2% BSA. Cells were collected and analyzed by flow cytometry. The percentage of phagocytosis was evaluated by comparing the ratio of double positive macrophages (CFSE^+^RFP^+^) with the total number of macrophages (CFSE^+^).

### Polarization of M2 macrophages to M1 macrophages

RAW 264.7 macrophages were plated in 12-well plates at 1 × 10^5^ cells/well for overnight. Subsequently, PBS, PQ912, BSA& Tetra-PEG-SS and PQ912+ BSA& Tetra-PEG-SS was dissolved in sterile CBS and DMEM equal volume mixture respectively to simulate the PB-Gel composition. The cells were then treated with the solution for 48 h. After the cells were treated with the above solution for 48 h, the cells were washed with PBS, centrifuged, and incubated with CD80 (Biolegend, cat. no.104721) and CD206 (Biolegend, cat. no.141704) for 30 min. The cells were quantified using BD FACSCalibur flow cytometry and analyzed using FlowJo software.

### Detection of CD47 expression on tumor cells

B16F10 cells were placed in 12-well plates with a cell count of 1 × 10^5^ per well and incubated overnight. Cells that adhered to the wall were treated with PBS and PQ912 for 48 h. The cells were then stained with PE-labeled CD47 antibody (clone miap301) at 4 °C for 30 min. After that, rinsed with PBS for 3 times and analyze by BD FACSCalibur flow cytometry. Mean fluorescence intensity (MFI) indicated the relative expression of CD47 on the surface of B16F10 cells.

### Postoperative recurrence model and treatment of orthotopic B16F10 tumor

To investigate the therapeutic effect of anti-melanoma recurrence after operation, 2 × 10^6^ B16F10-luc cells were transplanted into the right flanks of female C57BL/6 mice (6 to 8 weeks) on day 0. After 6 days, the tumors were removed leaving about 1% of residual tumors, to simulate the presence of residual microtumors on the clinical surgery.

Simply put, mice were anesthetized by intraperitoneal injection of chloral hydrate (400 mg/kg) and about 99% of the tumors were removed using sterile instruments. Then, the mice were randomly divided into four groups (n = 8) and received the following treatments immediately: (i) no treatment will be done (Untreated), (ii) PB-Gel (100 μL), (iii) PQ/PB-Sol (a mixed solution of PQ912, BSA and Tetra-PEG-SS) (100 μL, 1mg PQ912 per mouse), (iv) PQ/PB-Gel (100 μL, 1mg PQ912 per mouse). The wound was then closed with absorbable surgical sutures. Tumor bioluminescence imaging was obtained 10 minutes after intraperitoneal injection of D-luciferin potassium dissolved in DPBS (15 mg/mL, 10 μL/g) using the IVIS spectrophotometric imaging system on the day before surgery and every five days after surgery. Because testing for a triggered antitumor immune response *in vivo* required killing a batch of mice on a specified number of days to obtain a sample, survival studies required purchasing a second batch of mice and randomly assigning eight mice to each group. The survival time of the model mice was recorded from the day of tumor cell inoculation, and the survival curve was drawn.

### In vivo immune response analysis

Tumors in each group were divided into small same volume pieces, which were digested by digestive enzymes at 37 ℃ for 2 h. And then all the samples by nylon mesh filter (40 μm) were ground into single-cell suspension. Erythrocytes were removed and lymphocytes were collected using mouse lymphocyte separator kit. Cells were stained with different fluorescence-labeled antibodies following the manufacturer's instructions. The stained cells were measured on BD FACS Calibur flow cytometry and analyzed by the FlowJo software.

Tumor and spleen infiltrating lymphocytes were extracted using by infiltrating tissue lymphocyte separation solution kit followed the manufacturer instructions. Cells were stained with different fluorescence-labeled antibodies following the manufacturer's instructions. The membrane was fixed and broken with intracellular fixation and osmotic buffer. The stained cells were measured on BD FACSCalibur flow cytometry and analyzed by the FlowJo software.

### The exploration of the time when immune function is strongest

To explore the best time for tumor resection and treatment, 1 × 10^6^ B16F10-luc cells were transplanted into the left side of the abdomen of female C57BL/6 mice (6 to 8 weeks). The primary tumors were partially removed and the PQ/PB-Gel was *in situ* covered into the surgical tumor bed after 3, 6 and 9 days respectively (n = 3). Another tumor, called a proximal tumor (1 × 10^6^ B16F10-luc cells), was inoculated on the right wing of the abdomen for each mouse after 3 days. Tumor development was observed using IVIS spectrophotometric imaging system on the 3th, 6th day after proximal tumor inoculation.

To explore how long after administration of the drug, immune function *in vivo* is at its peak, we transplanted 1 × 10^6^ B16F10-luc cells into the left abdomen of female C57BL/6 mice (6 to 8 weeks). The primary tumor was then partially resected and PQ/PB-Gel was *in situ* covered into the surgical tumor bed on the 5th postoperative day (n = 3). The mice were euthanized at 3, 6 and 9 days after administration, and the memory cells in spleen were analyzed by flow cytometry.

### Inhibitory effect of memory cells on newly formed tumor

Six days after 1 × 10^6^ B16F10-luc cells were transplanted into the back of female C57BL/6 mice (6-8 weeks), part of the primary tumor was resected and PQ/PB-Gel was *in situ* covered into the surgical tumor bed. The untreated group served as the control group. Three days later, another tumor was inoculated in the abdomen of each mouse as a distal tumor. Tumor bioluminescence imaging was performed using the IVIS spectrophotometric imaging system at days 5, 6, 12, and 15.

The formation of memory cells in the distal model was analyzed by flow cytometry. In brief, peripheral blood mononuclear cells of each group were isolated by incubating with RBC lysing buffer. Cells were stained with different fluorescence-labelled antibodies following the manufacturer's instructions.

### Therapeutic effect of PQ/PB-Gel on unoperated mouse tumor model

To investigate the therapeutic effect of PQ/PB-Gel on unoperated tumor-bearing mice, 1 × 10^6^ B16F10-luc cells were transplanted into the left abdomen of female C57BL/6 mice (6 to 8 weeks). On the third days after tumor inoculation, PQ/PB-Gel was applied *in situ* to the tumor site. Another type of tumor (1 × 10^6^ B16F10-luc cells) was inoculated on the right side of the abdomen of each mouse after 3 days. The development of the tumor was observed by IVIS spectrophotometric imaging system on days 3, 6, 9 and 12 and the survival time of model mice was recorded to draw the survival curve.

### Postoperative recurrence model and treatment of ectopic 4T1 tumor

To investigate the therapeutic effect of cold tumor recurrence after surgery, 4T1 cells (5 × 10^6^ cells) were inoculated subcutaneously into the right flank of female BALB/c mice (6 to 8 weeks of age) on day 0. The mice were randomly divided into six groups (n = 8) and immediately given the following treatments: (i) no treatment will be done (Untreated), (ii) PB-Gel (100 μL, injected *in situ*), (iii) PQ/PB-Sol (100 μL, 1mg PQ912 per mouse, injected *in situ*) (iv) aPD-1 (2.5 mg/kg, intravenous injection), (v) PQ/PB-Gel (100 μL, 1mg PQ912 per mouse, injected *in situ*). aPD-1 was injected on days 6, 9, 12, and 15 via the tail vein. One day before surgery and every five days after surgery, potassium D-luciferin dissolved in DPBS (15 mg/mL, 10 μL/g) was intraperitoneally injected, and tumor bioluminescence imaging was performed using the IVIS spectrophotometric imaging system. The tumor growth curve was drawn by calculating the change of bioluminescence intensity over time, and the survival time of model mice was recorded to draw the survival curve.

### Comparison of anti-relapse efficacy between PQ/PB-Gel and aCD47 antibody

The construction methods of ectopic 4T1 tumor and orthotopic B16F10 tumor models were the same as described above. In the 4T1 model, the mice were randomly divided into four groups (n = 7) and immediately given the following treatments: (i) no treatment will be done (Untreated), (ii) aCD47(50 μg aCD47 per mouse, intravenous injection), (iii) aCD47/PB-Gel (100 μL, 50 μg aCD47 per mouse) (iv) PQ/PB-Gel (100 μL, 1mg PQ912 per mouse). In the B16F10 models, the mice were randomly divided into four groups (n = 6) and immediately given the following treatments: (i) no treatment will be done (Untreated), (ii) aCD47(50 μg aCD47 per mouse, intravenous injection), (iii) aCD47/PB-Gel (100 μL, 50 μg aCD47 per mouse) (iv) PQ/PB-Gel (100 μL, 1 mg PQ912 per mouse). aCD47 was injected intravenously on days 6, 9, 12, and 15 via the tail vein. One day before surgery and every five days after surgery, potassium D-luciferin dissolved in DPBS (15 mg/mL, 10 μL/g) was intraperitoneally injected, and tumor bioluminescence imaging was performed using the IVIS spectrophotometric imaging system. The tumor growth curve was drawn by calculating the change of bioluminescence intensity over time, and the survival time of model mice was recorded to draw the survival curve. Two weeks later, the mice were euthanized and whole blood was collected to detect the number of RBCs and hemoglobin (HGB).

### Cytokines

Serum of each group (Untreated, PB-Gel, PQ/PB-Sol, PQ/PB-Gel, aCD47, aCD47/PB-Gel) of C57BL/6 mice was taken as test samples, and CBA detection technology was used to detect the expression of each factor in the samples.

### Immunofluorescence staining

Tumors from mice were immobilized in 4% paraformaldehyde for 48 h and then metastasized into 75% alcohol. The thin sections of 3 μm were obtained by microtome and stained with different primary antibodies: CD8 (Thermo Fisher Scienctific, cat. no. MA1-81180), CD80 (Abcam, cat. no. ab254579), and F4/80 (Cell Signaling technology, cat. no. 71299S), CD47 (ABclonal, cat. no. A7278) overnight at 4 °C following the manufacturer instructions. Following the addition of fluorescently labelled secondary antibodies (goat anti-rat IgG (H&L; Abcam, cat. no. ab150159) and goat anti-rabbit IgG (H&L; Abcam, cat. no. ab150077)), the slides were analyzed with a confocal microscope. The antibodies used in the experiment were diluted according to instructions.

### Statistical analysis

Statistics were performed on GraphPad Prism 7. All data were presented as the means value ± SD. Statistical analysis was performed using Student's t test (two-tailed) and one-way analysis of variance (ANOVA). Statistical significance was established at *P* < 0.05, where *P < 0.05, **P < 0.01, ***P < 0.001 and ****P < 0.0001.

## Results

### In situ formation of biodegradable gel by two-component syringe

PEG-based hydrogels have a wide range of applications in medical devices and regenerative medicine, especially in sustained or controlled drug release, two-dimensional and three-dimensional cell culture, and wound suture and healing. Commonly used multi-arm PEG materials included two arm-, four arm-, eight arm- PEG. Previous studies have reported that the introduction of cyclized succinyl groups (succinimidyl succinate) into the hydrogel matrix makes the hydrogel have the ability of rapid degradation and hemostasis, showing good biocompatibility. We compared the internal structure, *in vivo* and *in vitro* release and swelling of PB-Gel-2, PB-Gel-4 and PB-Gel-8 formed by three kinds: 2 arm-PEG-SS, Tetra-PEG-SS and 8 arm-PEG-SS with BSA respectively. In order to detect the release behavior of small-molecule immunomodulators in hydrogels, the fluorescent dye rhodamine B (Rom) was used as the model drug, and the fluorescence intensity was monitored by the fluorescence IVIS. As shown in Figure S1, the pore size of PB-Gel-2 was larger, and the release rate of simulated drugs (Rom and PQ912) from PB-Gel-2 was faster *in vivo* and *in vitro*, and the swelling rate was larger. On the contrary, PB-Gel-8 had a small pore size, and the release rate of drugs from PB-Gel-8 was slow and the swelling rate was small. PB-Gel-8 had a small swelling rate, but after loading PQ912, the cumulative release rate of PQ912 could only reach about 59.63% *in vitro*. According to comprehensive analysis, the properties of PB-Gel-4 were between PB-Gel-2 and PB-Gel-8, which could ensure the effective and continuous release of drugs without large swelling, fitting well with the narrow cavity to avoid the additional strain. Therefore, PB-Gel-4 (PB-Gel was used in the following) was selected as the injectable hydrogel carrier for postoperative immunotherapy.

**Figure 2:**
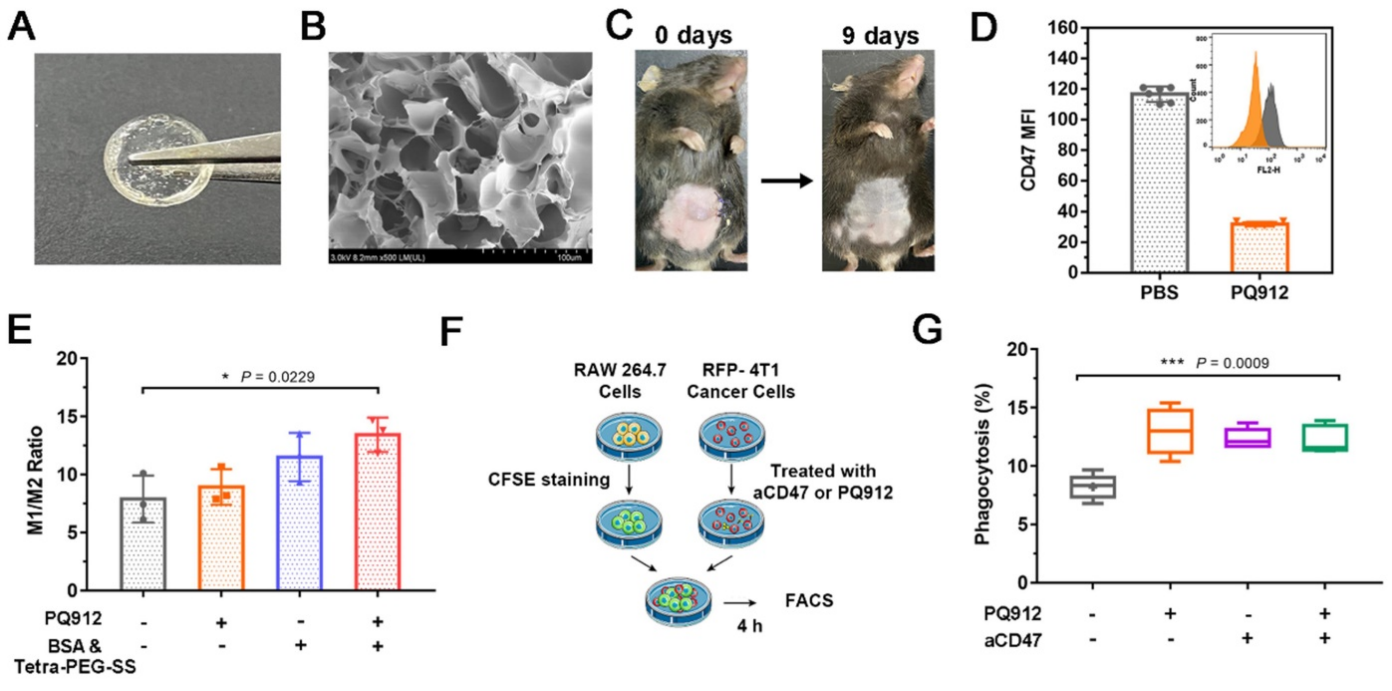
Characterization of *in situ* small molecule hydrogel and *in vitro* efficacy studies of PQ/PB-Gel. (A) Picture of PQ/PB-Gel. (B) Representative scanning electron microscope images of PB-Gel. (C) Image of PB/Gel degradation *in vivo*. (D) The expression of CD47 in B16F10 cells was detected by flow cytometry. Data shown as means ± SD (n = 6). (E) Graph demonstrating expression of M1/M2 ratio in TAMs was quantitatively detected by flow cytometry 48 h after different treatments. Data shown as means ± SD (n = 3). *P < 0.05, **P < 0.01, and ***P < 0.001 and ****P < 0.0001 by one-way analysis of variance. (F) Schematic diagram of phagocytosis assay. The RFP-4T1 cancer cells were incubated with different treatments for 24 h. Subsequently, RAW264.7 macrophages were stained with CFSE and co-cultured with treated RFP-4T1 cells, and flow cytometry was performed. (G) Graph showed the phagocytosis percentage of RAW264.7 macrophages co-cultured with 4T1 breast cancer. Data shown as means ± SD (n = 3). *P < 0.05, **P < 0.01, ***P < 0.001 and ****P < 0.0001 by one-way analysis of variance.

As shown in Figure S2A, Tetra-PEG-SS and BSA were rapidly cross-linked under alkaline conditions through a mixer at the front of a two-component syringe to form a hydrogel (PB-Gel). The gelation time was optimized, the resulting system at pH 10 polymerized in 10 s when injected, providing rapid secure protection and reduced adhesion (Figure S2B). As shown in Figure 2A, B, the morphology of the transparent hydrogel scaffold under scanning electron microscope showed a porous network. Meanwhile, it was biodegraded in about 10 days and allowed for the sustained release of PQ912 (Figure 2C). The rheological test results further proved that the hydrogel network structure had a long relaxation time (Figure S2C). This was consistent with the observation that the hydrogel could maintain its shape without flowing in the inverted glass bottle.

### PQ912 induces tumor cell death indirectly through down-regulating the CD47 expression

To assess the antitumor effect of PQ912, we firstly determined its direct effect on tumor cells. PQ912 showed no significant cytotoxicity to 4T1 and B16F10 tumor cells even at concentrations up to 50 μM after 24 h incubation (Figure S3A and S3B). Next, we detected the changes of CD47 in PQ912-treated mouse B16F10 cells using PE-labeled anti-mouse CD47 Antibody (Clone: MIAP301). Compared with the control group, the expression of CD47 on the surface of B16F10 cells treated with PQ912 decreased by about 3.65 times (Figure 2D), indicating the potential of PQ912 in blocking the CD47-SIRPα axis by down-regulating the expression of CD47.

### Hydrogels enhance the recognition and phagocytosis of macrophages to tumor antigens through immune pathways

In order to study the intervention effect of the constructed hydrogel scaffold on immune regulation, we studied the effect of hydrogel components on macrophage type *in vitro*. As shown in Figure 2E, both the weakly basic hydrogel matrix (BSA& Tetra-PEG-SS) and PQ912 could promote the polarization of TAMs to M1 type, and the effect became more significant when treated together. Then, we co-cultured the CFSE (carboxyfluorescein succinimidyl amino ester)-stained RAW 264.7 with aCD47- or PQ912-treated 4T1 cells expressing red fluorescent protein (RFP-4T1) to investigate the phagocytosis rate of macrophages (Figure 2F). As exhibited in Figure 2G, treating tumor cells with either PQ912 or aCD47 could significantly promote the phagocytosis of macrophages to tumor cells, indicating that PQ912 can play the same effect as aCD47. However, no synergistic effect was observed in the combination with PQ912 and aCD47. All the data suggested that the effect of PQ912 might be ascribed to the regulation of CD47 expression on the tumor cell surface rather than directly killing tumor cells.

### Local slow release of immunomodulators can prevent B16F10 melanoma recurrence

As shown in Figure S4A, in mice treated with Rom dispersed in the same volume PBS containing a mixture of BSA and Tetra-PEG-SS (Rom/PB-Sol), the fluorescence intensity at the site of operating surgery decreased significantly after 1 day. In contrast, the fluorescence intensity of the implant site treated with PB-Gel loaded Rom (Rom/PB-Gel) decreased slowly within 8 days and near disappeared in 10 days (Figure S4 A, B).

**Figure 3:**
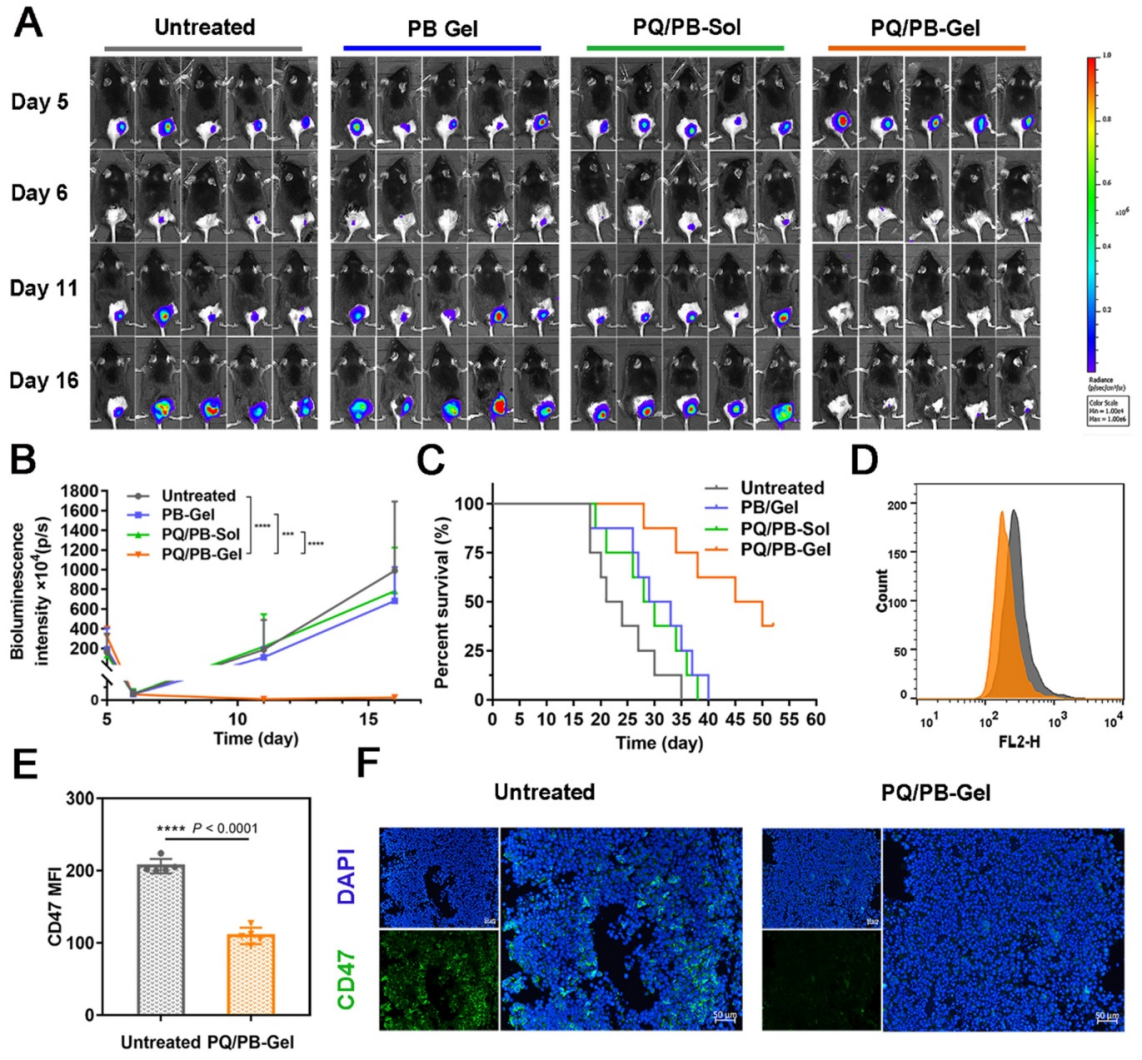
PQ/PB-Gel for reducing recurrence of B16F10 melanoma after surgery and exerting antitumor immune responses *in vivo*. (A) Bioluminescence images of recurrent tumors in mice treated with different treatments before and after primary tumor resection. (B) Quantitative curve of tumor growth in each group at days 5, 6, 11,16 from tumor inoculation. Data are presented as means ± SD (n = 6). (C) The survival rate of mice after different treatments (n = 8). (D) Flow cytometric histogram of the expression of CD47 in tumor cells. (E) MFI of CD47. Data are presented as means ± SD (n = 5). (F) Representative immunofluorescence of tumors exhibited CD47^+^. Scale bar, 50 μm. Data are presented as means ± SD (n = 5). *P < 0.05, **P < 0.01 ***P < 0.001 and ****P < 0.0001 by two-tailed Student's t test.

Given that the release of these immunomodulators could be prolonged locally *in vivo*, we next intended to evaluate the benefit of the hydrogel against the tumor recurrence. Since it was difficult to distinguish the tumor from the gel matrix by measuring tumor volume with a vernier caliper, the bioluminescence signal of B16F10 cells detected by IVIS was used to monitor the tumor growth. Preliminary experiment demonstrated that the favorable treatment time after tumor inoculation was between 3 and 6 days (Figure S5). In order to distinguish the subsequent treatment effect, we chose to implement the treatment on the 6th day after the inoculation. As shown in Figure 3A, 3B, compared with the other three groups, mice treated with PQ912 hydrogel (PQ/PB-Gel) showed almost no tumor growth at the tumor resection cavity on day 5 postoperatively (the masses seen are not fully degraded gel matrix due to their soft and elasticity) and tumor growth was still effectively controlled on day 10 postoperatively. After 40 days of tumor inoculation, the survival rate of mice treated with PQ/PB-Gel could still reach 62.5%, while all mice in other groups had died (Figure 3C). To verify the mechanism, the residual tumors were collected at 12 days after surgery and analyzed by flow cytometry and immunofluorescence staining. As expected, CD47 expression quantity on the tumor cell surface was downregulated *in vivo* with the slow release of PQ912 (Figure 3D- F).

### Innate immune cells mobilize adaptive immune cells to work together to prevent the regeneration of recurrent B16F10 melanoma

**Figure 4:**
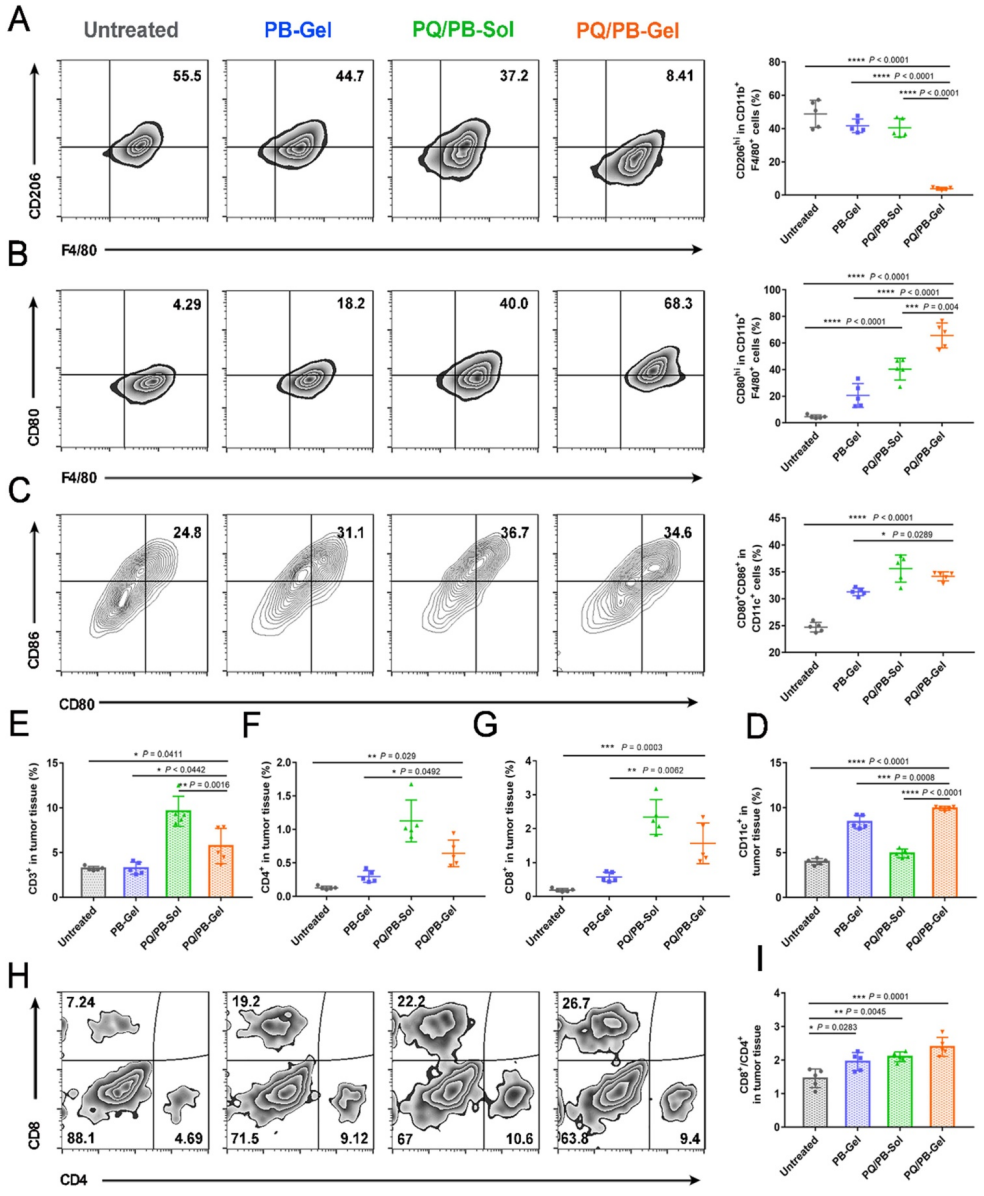
PQ/PB-Gel for triggering antitumor innate and adaptive immune response after B16F10 melanoma surgery. (A and B) Representative flow cytometric images (left) and relative quantitative images (right) of M2-like TAMs (CD206^hi^) and M1-like TAMs (CD80^hi^) gating on F4/80^+^CD11b^+^CD45^+^ cells. Data are presented as means ± SD (n = 5). (C) Representative flow cytometric images (left) and relative quantification images (right) of CD80^+^CD86^hi^ DCs gating on CD11c^+^ cells. Data are presented as means ± SD (n = 5). (D) Relative quantitative images of CD11c^+^ cells. Data are presented as means ± SD (n = 5). (E) Relative quantification of CD3^+^ cells within the tumor. Data are presented as means ± SD (n = 5). (F and G) The quantification results of CD4^+^, CD8^+^ cells within the tumor in different groups. Data are presented as means ± SD (n = 5). (H) Representative flow cytometric analysis of T cells infiltration within the tumor gating on CD3^+^ CD45^+^ cells. (I) The ratio of CD8 to CD4 cells within the tumor in different groups. Data are presented as means ± SD (n = 5). *P < 0.05, **P < 0.01, ***P < 0.001 and ****P < 0.0001 by one-way analysis of variance.

To explore the immune response after local administration, the tumors were harvested at 12 days post-surgery for immunophenotyping. Compared with the other three groups, the proportion of M2-like TAMs (CD206^hi^F4/80^+^CD11b^+^) in the PQ/PB-Gel group was remarkably reduced (Figure 4A). Meanwhile, compared with the untreated group, the proportion of M1-like tumor-associated macrophages (M1-like TAMs) (CD80^hi^F4/80^+^CD11b^+^) in the tumor microenvironment (TME) of the other three groups increased, and the significant shift was observed in the treatment of the PQ/PB-Gel group (Figure 4B). The immunofluorescence intensity of CD80^+^F4/80^+^ cell in the center and periphery of B16F10 tumor tissue after PQ/PB-Gel treatment was significantly higher than that of untreated group (Figure S6). As also shown in Figure 4C, 4D, it was found that the tumor treated by the PQ/PB-Gel group contained high percentage of mature CD11c^+^ dendritic cells (DCs), which expressed CD80 and CD86.

Since previous studies demonstrated the potential of the activation of antigen-presenting cells and the stimulation of T cell-mediated antitumor immunity via the blocking of the interaction between CD47 and SIRPα, we next characterized the adaptive immune cell subsets upon the treatment. As shown in Figure 4E, the relative proportion of CD3^+^ tumor infiltrating lymphocytes after PQ912 treatment was significantly higher than those of the other groups. We observed a significant increased percentage of CD4^+^ T cells (helper T cells) and CD8^+^ T cells in TME upon PQ912 treatment (Figure 4F, 4G). What's more, after PQ/PB-Gel treatment, the infiltration of CD8^+^ T cells increased both in the center and the periphery of the tumor tissue (Figure S7). Collectively, the adaptive immune response that activated CD8^+^ T cells to differentiate into cytotoxic T cells (CTLs) might be partially responsible for the efficacy of local PQ/PB-Gel (Figure 4H, 4I).

### PQ/PB-Gel remodels the immunosuppressive TME at the tumor resection cavity

Immunosuppressive TME plays a negative role in tumor immunotherapy, so we examined the effect of PQ/PB-Gel on the immunosuppressive TME to further understand their immunoregulatory potential 19. After PQ/PB-Gel treatment, the levels of regulatory T cells (Tregs) in TME decreased slightly (Figure S8A). But a significant reduction was observed in myeloid derived inhibitory cells (MDSCs) after treatments as compared with the untreated group (Figure S8B). Notably, the relative proportion of CD45^+^ cells in the PQ/PB-Gel group was close to that of the untreated group, but the proportion of MDSCs in CD45^+^ cells was observed to be much lower, indicating the inhibitory effect of PQ/PB-Gel upon MDSCs (Figure S8C). Secretion of cytokines including interleukin 10 (IL-10), interleukin12p70 (IL-12p70) and interferon-γ (IFN-γ) further confirmed the effective innate and adaptive immune responses induced by PQ/PB-Gel treatment (Figure S9).

### PQ/PB-Gel induces immune response and memory to improve cancer prognosis

**Figure 5:**
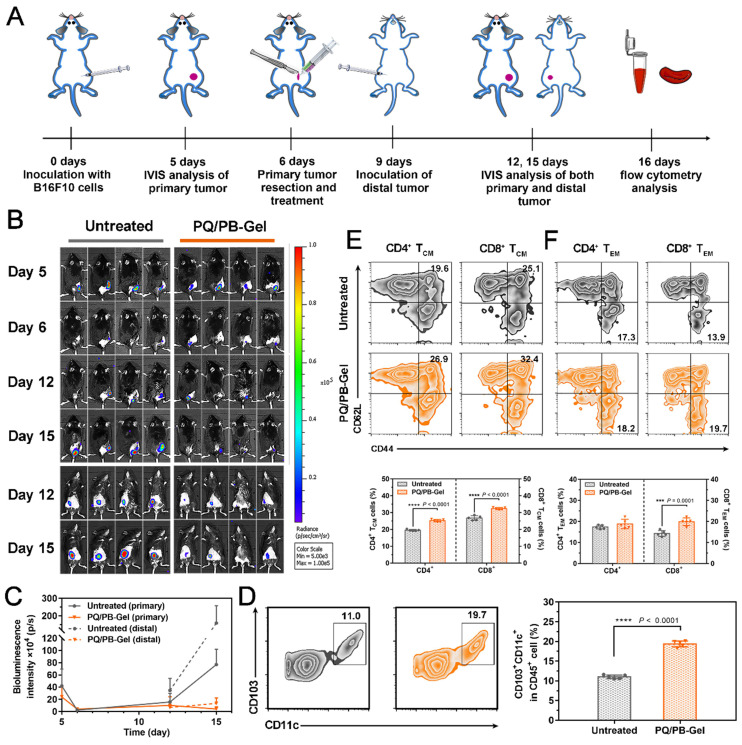
PQ/PB-Gel induces immune memory effect of B16F10 tumor suppression. (A) Diagram of treatment schedule. Method of treatment: (i) no treatment will be done (Untreated), (ii) PQ/PB-Gel (100 μL, 1mg PQ912 per mouse, injected *in situ*). (B) Bioluminescence images of distal tumor growth before and after primary tumor resection in mice treated with different treatments. (Top: an *in situ* tumor of the back; Bottom: a distal tumor of the abdomen) (C) Primary and distal tumor growth curves of different treatment groups at 5, 6, 12 and 15 days after tumor inoculation. Data are expressed as means ± SD. (n = 6). (D) Representative flow cytometric images of CD103^+^CD11c^+^ DCs gating on CD45^+^ cells (left) and relative quantitative images (right). Data are presented as means ± SD (n = 5). (E) Representative flow cytometric images of CD4^+^ and CD8^+^ T_CM_ cells (CD44^+^CD62L^+^) in spleens of mice of different treatment groups gating on CD3^+^ cells (top) and relative quantitative images (bottom). Data are presented as means ± SD (n = 5). (F) Representative flow cytometric images of CD4^+^ and CD8^+^ T_EM_ cells (CD44^+^CD62L^-^) in blood of mice of different treatment groups gating on CD3^+^ cells (top) and relative quantitative images (bottom). Data are expressed as means ± SD (n = 5). *P < 0.05, **P < 0.01, ***P < 0.001 and ****P < 0.0001 by two-tailed Student's t test.

Considering the presence of micro-metastases and/or scattered tumor cells in the circulation of most patients received surgery, the ability of PQ/PB-Gel group via local immunotherapy in preventing local and distant recurrence was explored. Correspondingly, we re-inoculated tumor distally to simulate metastatic tumor growing in mice received primary tumor excision and monitored the growth of the distal tumor (the protocol depicted in Figure 5A). Compared to the untreated group, the mice treated with PQ/PB-Gel had almost no local tumor recurrence and minimized distal tumor growth (Figure 5B, 5C). In order to verify the mechanism, the infiltration of DCs in the tumor tissue was examined and the result showed increased relative content of CD103^+^ DCs after PQ/PB-Gel treatment (Figure 5D). After activation and antigen processing, CD8^+^ T cells partially differentiated into memory T cells, which could be further divided into central memory T (T_CM_) cells and effector memory T (T_EM_) cells. Local administration of PQ/PB-Gel resulted in enhanced immune memory function of spleen in a time-dependent manner, and reached the highest level after the hydrogel was completely degraded (the 9th day) (Figure S10), which had not been explored in previous studies. In terms of functionality, T_EM_ cells could quickly play the role of fighting against tumor cells. As for the T_CM_ cells, they would be expanded when it is re-exposed to the cognate antigen, providing the resulting systemic anticancer immune response against distant tumor growth over a longer period of time 27. At the end of the treatment cycle, the percentages of T_CM_ cells and T_EM_ cells in spleen and CD8^+^ T_EM_ cells in blood of mice treated with PQ/PB-Gel were significantly increased as compared with the untreated group, while the percentages of CD4^+^ T_EM_ cells in blood remained unchanged (Figure 5E, 5F, and Figure S11) 20. All the data suggested that the development of long-term immune memory delayed the metastatic tumor. It was possibly due to the combination of continuously released immunostimulant and the residual damaged tumor cells acting as* in situ* vaccine antigen, resulting in activated antitumor immune cells and induced memory cells against B16F10 tumor.

We further tested the potency of PQ/PB-Gel alone or in combination with aPD-1 in direct inhibition of B16F10 tumor. However, in the absence of surgical protocols, treatment of PQ/PB-Gel alone showed mild inhibition of tumor growth as compared with the untreated group, but no synergistic effect was observed in the combination with aPD-1 (Figure S12). This might be due to the formation of a “protective net” around the undamaged tumor tissue, where immune cells located at the edge of the tumor site (invasive margin), preventing deep penetration of therapeutic substances 21. Most immunomodulators could only temporarily interfere with the environment around the tumor tissue, and only a few could elicit a weak tumor immune response. Therefore, for “cold tumor” that also lack the infiltration of immune cells, the physical barrier can be removed by surgery. PQ/PB-Gel can be administered *in situ* to fight against the immunosuppressive environment and induce subsequent innate and adaptive immune responses, so as to achieve a better therapeutic effect.

### PQ/PB-Gel can effectively inhibit tumor recurrence for “cold” tumors

**Figure 6:**
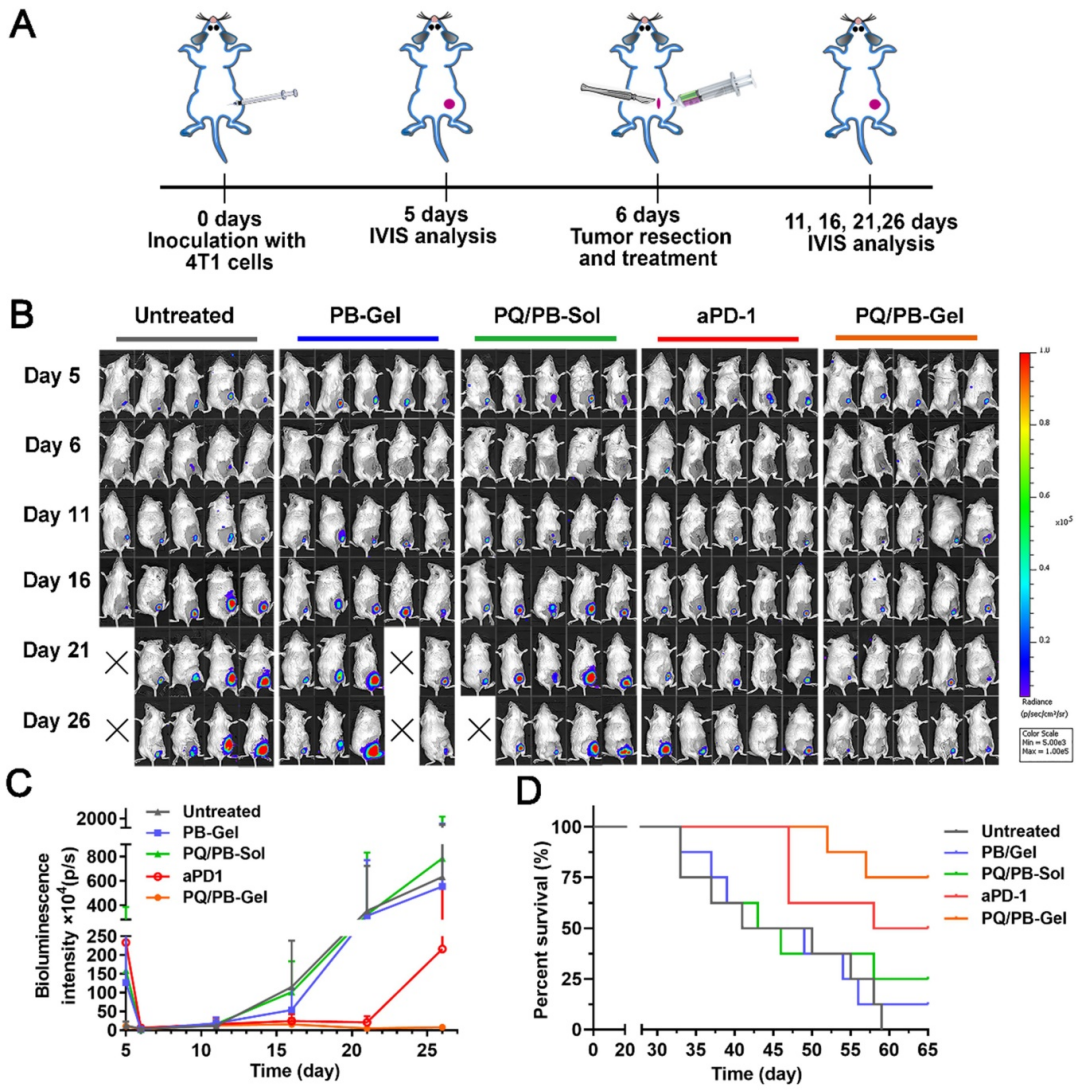
The inhibition effect of PQ/PB-Gel alone on “cold” tumor (4T1 tumor) postoperative recurrence was stronger than that of aPD-1 alone. (A) Diagram of treatment schedule. Method of treatment: (i) no treatment will be done (Untreated), (ii) PB-Gel (100 μL, injected *in situ*), (iii) PQ/PB-Sol (100 μL, 1mg PQ912 per mouse, injected *in situ*), (iv) aPD-1 (2.5 mg/kg, intravenous injection), (v) PQ/PB-Gel (100 μL, 1mg PQ912 per mouse, injected *in situ*). (B) Bioluminescence images of 4T1 ectopic tumor growth before and after resection of primary tumor in mice treated with different methods. (C) Growth curves of 4T1 tumor in different treatment groups at 5, 6, 11, 16, 21, and 26 days after tumor inoculation. Data were expressed as mean ± SD (n = 6). (D) Survival rate of mice after different treatments. (n = 8).

On the basis of the above existing studies, we further explored the effect of PQ/PB-Gel on the 4T1 “cold” tumor recurrence model. The absence of T cell infiltration may explain the limited response of ICIs as monotherapy (response rates in the range of 10-35%) 22, 23. It is well known that ICIs therapy is generally more sensitive to “hot” tumor. However, “cold” tumor generally has a low tumor mutation burden and T cells infiltration, so ICIs therapy in the absence of an adaptive immune response to treat “cold” tumor is a challenge 24-26. Previous reports showed that by breaking tolerance, ICIs allowed the release of a pre-existing immune response to reject tumors 27. Inspired by this, we tried to change TME through PQ/PB-Gel, with the mobilization of innate immune response as the starting point, to provide more T cells infiltration for TME. As illustrated in Figure 6A, compared with the untreated group, treatment with aPD-1 alone after surgery tended toward delay 4T1 tumor recurrence and progression, but the effects did not reach the statistical significance. Surprisingly, the group treated with PQ/PB-Gel alone resulted in effectively controlling tumor growth within 25 days, and the therapeutic effect on “cold” tumor was much better than that of aPD-1 alone (Figure 6B, 6C). As showed in Figure 6D, the survival rate at 60 days after PQ/PB-Gel treatment was 75%, which was significantly higher than that in aPD-1 group (50%), indicating that PQ/PB-Gel could effectively inhibit tumor recurrence for “cold” tumor.

We focused on the infiltration of tumor T cells. Many literatures have reported that aPD-1 therapy didn't improve T cells infiltration well. While it was gratified that the treatment of PB-Gel and PQ/PB-Gel could effectively improve the plight of fewer CD4^+^ (Figure S13A) and CD8^+^ T cells (Figure S13B) in “cold” tumor infiltration, respectively. As illustrated in Figure S13C, similar to the B16F10 melanoma model, PQ/PB-Gel significantly down-regulated the expression of CD47 on the surface of 4T1 tumor cells. We then examined the reversal of the immunosuppressive microenvironment in the “cold” tumor model. As shown in Figure S13D, The PB-Gel treatment group had an obvious effect on the down-regulation of M2-like TAMs. Compared with untreated group, aPD-1 and PQ/PB-Gel treatment group could effectively reduce MDSCs in “cold” TME (Figure S13E). In summary, PQ/PB-Gel treatment group not only had a better effect than aPD-1 in reversing the immunosuppressive microenvironment of “cold” tumor, but also could effectively improve T cell infiltration in “cold” tumor compared with ICIs of aPD-1.

### PQ/PB-Gel shows stronger antitumor recurrence effect than aCD47

**Figure 7:**
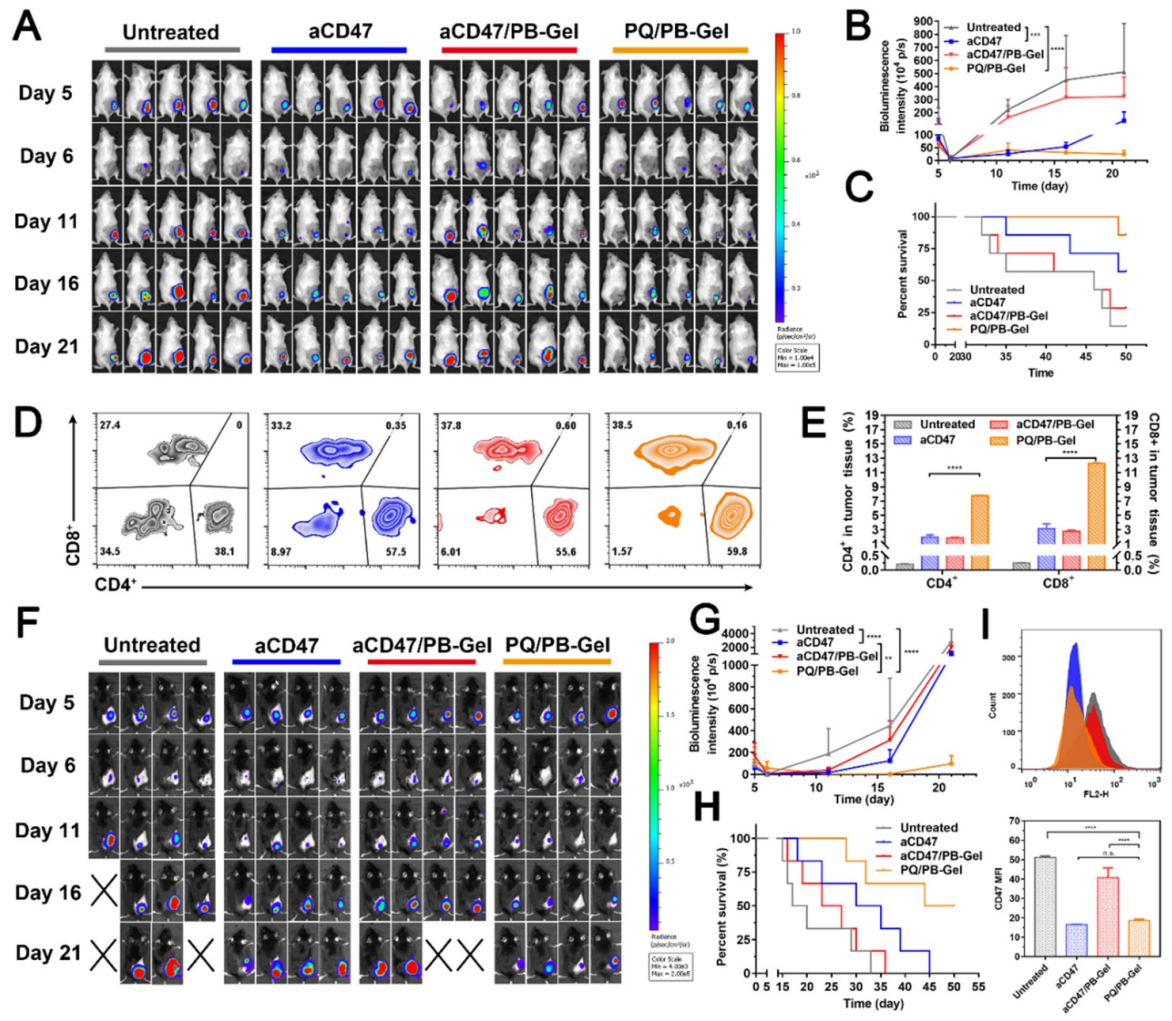
Inhibitory effect of PQ/PB-Gel and aCD47 antibody on postoperative recurrence of 4T1 and B16F10 tumors. (A) Bioluminescence images of 4T1 ectopic tumor growth before and after resection of primary tumor in mice treated with different treatments. (B) Growth curves of 4T1 tumor in different treatment groups at days 5, 6, 11, 16 and 21 after tumor inoculation. Data were expressed as means ± SD (n = 5). (C) Survival rate of mice after different treatments against 4T1 tumor (n = 7). (D) Representative flow cytometric analysis of T cells infiltration within the tumor gating on CD3^+^CD45^+^ cells. (E) Relative quantitative images (right) of CD4^+^ and CD8^+^ T cells gating on CD3^+^CD45^+^ lymphocyte^+^ cells. Data are presented as means ± SD (n = 5). (F) Bioluminescence images of B16F10 tumor growth before and after primary tumor resection in mice treated with different treatments. (G) Quantitative curve of B16F10 tumor growth in each group at days 5, 6, 11,16 and 21 from tumor inoculation. Data are presented as means ± SD (n = 4). (H) The survival rate of mice after different treatments. (n = 6). (I) Flow cytometric of the expression of CD47 in tumor cells (top) and histogram of MFI of CD47 (bottom). *P < 0.05, **P < 0.01, ***P < 0.001 and ****P < 0.0001 by two-tailed Student's t test.

To explore whether PQ/PB-Gel can achieve the same therapeutic effect as aCD47, we set up two positive control groups: intravenous injection of aCD47 and loading of aCD47 into PB-Gel (aCD47/PB-Gel). As illustrated in the Figure 7A, 7B, mice intravenously given aCD47 alone showed tumor recurrence progressed slowly, however, disease worsened after 16 days. At 21 days after 4T1 tumor inoculation, there was almost no tumor recurrence in the PQ/PB-Gel group. Compared with aCD47 and PQ/PB-Gel groups, aCD47/PB-Gel group had a poor anti-recurrence effect, and the survival was not significantly prolonged (Figure 7C). In addition, as shown in Figure 7D, 7E, tumor infiltrating lymphocytes (CD4^+^ and CD8^+^ T cells) in PQ/PB-Gel group were significantly increased compared with the other three groups. These results indicated that loading aCD47 into PB-Gel could not achieve a good synergistic immune effect. The similar phenomenon was observed in the B16F10 melanoma mode (Figure 7F- H). However, since melanoma is a rapidly proliferating malignant skin cancer, even mice treated with PQ/PB-Gel died after about 30 days. Nevertheless, compared with aCD47 and aCD47/PB-Gel groups, PQ/PB-Gel showed the best anti-melanoma recurrence effect, with a 50-day survival rate of 50%. Secretion of cytokines including IL-12p70, IFN-γ, and TNF-α further confirmed the effective innate and adaptive immune responses induced by PQ/PB-Gel were significantly stronger than those induced by aCD47 and aCD47/PB-Gel (Figure S14). This provided the possibility for future use in combination with chemotherapy drugs to completely inhibit melanoma recurrence. As shown in Figure 7I, by investigating the expression of CD47 on tumor surface, it was found that PQ/PB-Gel could reduce the expression of CD47 on tumor surface almost to achieve the same effect as aCD47 blocking.

### PQ912 can avoid the blood toxicity caused by aCD47 in terms of safety

**Figure 8:**
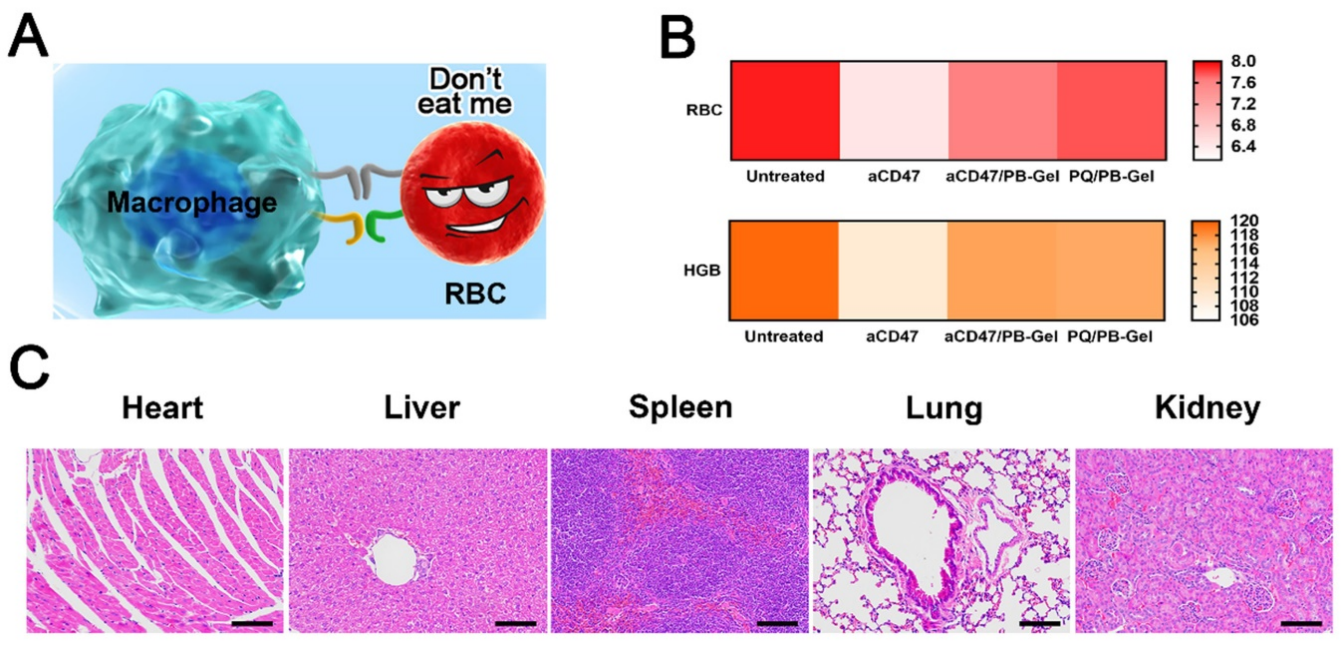
*In vivo* safety of PQ912. (A) PQ912 does not destroy the original CD47 on the RBCs. (B) Safety test in BALB/c mice after different treatments. The average number of RBCs (×10^12^/L) and HGB (×g/L) (C) H&E staining of major organs in the body after PQ/PB-Gel treatment.

aCD47 inevitably damaged RBCs while killing tumor cells 28. In clinical trials, how to protect RBCs while killing tumor cells to the maximum extent had become the key to the development of aCD47 (Figure 8A). Local immunotherapy improved local efficacy by increasing the bioavailability of PQ912 at the injection site, while limiting systemic exposure.

It was curious whether PQ912 didn't damage innocent RBCs. RBCs and HGB in the blood of mice after different treatments were used to evaluate the safety of the drug. It was found that the number of RBCs and HGB in the blood of aCD47-treated mice was slightly reduced compared with the control group, while there was no significant change after aCD47/PB-Gel and PQ/PB-Gel treatment (Figure 8B). H&E staining of the main organs in the body showed no organic damage after PQ/PB-Gel treatment (Figure 8C).

## Discussion

At present, surgical treatment is still the main clinical strategy for most solid tumor. Despite advances in surgical techniques, postoperative residual tumor cells may remain in the surgical margins or circulation, which would increase the risk of tumor recurrence and metastasis 29. Since the smallest residual lesion is more easily controlled than the primary tumor, immunotherapy during the postoperative period, when residual tumor is at its lowest level, may provide better therapeutic outcome 29, 30. The compartments and spaces provided by the surgical resection site facilitate the local delivery of immune-stimulating compounds at the tumor site, where the residual tumor cells could be used as the antigen source.

Tetra-PEG-SS provided reduced swelling profiles as compared with other PEG polymers, fitting well with the narrow cavity to avoid the additional strain. Moreover, the rapid degradation properties for PB-Gel reduced the risk of requiring mechanical debridement or surgical resection compared to commercial synthetic sealants (most of which are PEG-based, including Focalseal, Progel and CoSeal, widely used in anticoagulant patients) 31, 32. More importantly, the designed PB-Gel has been found to play a certain intervention role in the immune regulation: (i) the counteracting effect of weak alkaline system of PB-Gel on lactic acid and H^+^, which lead to impaired immune cell function, might help to improve immunosuppressive TME, (ii) the gel matrix promoted the activation of M1-like TAMs, and (iii) showed some reversal of the temporary immunosuppression associated with wound healing caused by the resection of the primary tumor 33.

Currently, three types of inhibitors of the CD47-SIRPα signaling pathway have been reported: molecules that inhibit pathway activity by blocking the CD47 molecules on target cells, molecules that inhibit pathway activity by blocking the SIRPα molecules on immune effector cells, and inhibitors of the QPCTL enzyme that is required for CD47 maturation. The aCD47 has been classified into two different mechanisms 34. Some CD47 antibodies such as B6H12 directly inhibit the interaction of CD47 with SIRPα to alter the specific function of CD47. While others including 1F7, AD22, MABL, and CC2C6 exhibit agonist or antagonist activity, possibly by altering aggregation or inducing conformational changes that alter the intrinsic intracellular signaling function of CD47. In contrast to aCD47, QPCTL inhibitors destroy the sites binding to SIRPα by inhibiting post-translational modification of CD47 protein (pyroglutamate modification of the amino terminal of CD47 protein). In addition, small molecule inhibitors of QPCTL have also been reported to manipulate the intensity of CD47-SIRPα signaling pathway 17. Unlike antagonists that bind to CD47 or SIRPα, CD47 molecules newly arriving on the cell surface under QPCTL inhibition (PQ912) lack the modification of pyroglutamate and therefore do not compete with natural binding partners in the TME 17. This means that, PQ912 will not destroy the original CD47 on the RBCs, avoiding the hematological side effects of CD47 antibodies, which provide hope for the safety of blocking the CD47-SIRPα signaling pathway.

One of the reasons why cancer vaccines can be given locally and have a long-term systemic effect is the production of initial specific antibodies and memory T cells. The key properties of memory T cells change over time after initiation, providing a unique environment for the innate cells at the time of revaccination. Like the antibody response, the T cell response is influenced by the number of antigenic encounters and varies over time. Compared with naïve cells, specific memory T cells respond are more frequent and faster, requiring fewer activation signals 35. Similar to the principle of local injection of vaccine, the local administration strategy of slowly and continuously releasing PQ912 to the tumor site treats the residual tumor cells as a natural antigen. This strategy may provide sufficient time and opportunity for the evolution of memory T cells and the times of contact with antigens. Therefore, as an ideal carrier of PQ912, PB-Gel hydrogel scaffold, which can play a certain intervention role in immune regulation, is expected to synergistically reverse immunosuppressive TME with PQ912 and provides the possibility for the generation of immune memory function.

In this study, the designed PQ/PB-Gel indirectly blocked and inhibited the “don't eat me” signal between tumor cells and macrophages, conducive to the recognition of tumor by innate immune cells. Activated dendritic cells and macrophages presented tumor antigens to adaptive immune cells and mobilized the adaptive immune cells to work closely to prevent the regeneration of B16F10 melanoma. After activation and antigen processing, CD8^+^ T cells partially differentiated into memory T cells, which could be further divided into T_CM_ cells and T_EM_ cells. Local application of PQ/PB-Gel induced a time-dependent enhancement of spleen immune memory function in mice, which had not been explored in previous studies. In terms of functionality, T_EM_ cells could quickly play the role of fighting against tumor cells. As for the T_CM_ cells, they would be expanded when it is re-exposed to the cognate antigen, providing the resulting systemic anticancer immune response against distant tumor growth over a longer period of time 36.

In our study, it was found that loading aCD47 into PB-Gel avoided hematological side effects caused by aCD47 alone, but the therapeutic effect was not ideal. In fact, to address the dilemma faced by anti-CD47 antibodies, nanodelivery strategies have been used in many preclinical studies to provide a solution to intervene in macrophage-mediated phagocytosis. Nie et al. synthesized a novel reactive exosome nanobiological conjugate that targeted both CD47 and SIRPα antibodies for the collaborative treatment of cancer 37. After systematic administration, exosomes could accumulate and release conjugated antibodies in tumor tissues by breaking benzoate-imine bond in acidic microenvironment, thus blocked the anti-phagocytosis signal and showed good antitumor effect. Chen et al. reported an *in situ* sprayed fibrin gel containing CaCO_3_ nanoparticles (aCD47@CaCO_3_) with aCD47 38. The results showed that the nanoparticle loaded gel system induced macrophages to phagocytize cancer cells by blocking the CD47-SIRPα interaction. In summary, we found that loading aCD47 into nanoparticles followed by other modifications showed antitumor activity. Therefore, we analyzed the poor anti-tumor effect of aCD47 directly loaded with PB-Gel. One possible reason was that the preparation process of aCD47/PB-Gel was the same as that of PQ/PB-Gel, and aCD47 was dissolved in weakly alkaline carbonate solution, which might lead to the destruction of its structure. In addition, the molecular weight of aCD47 was much higher than that of the small molecule PQ912, and the release rate of large molecules was slower in the hydrogel system. Therefore, aCD47/PB-Gel could not effectively inhibit the expression of CD47 at the tumor site in the early postoperative stage.

## Conclusions

To sum up, we have achieved the goal of reasonable combination of tumor surgical treatment and immunotherapy by *in situ* covering the surgically resected wound site with a two-component TME-remodeled hydrogel loaded with immunotherapy agent. In addition, the slow and continuous release of PQ912 effectively inhibited the generation of “don't eat me” signals during the treatment cycle, enhanced the clearance and recognition of cancer cells by macrophages and antigen-presenting cells, and further induced CTLs. More significantly, the local administration of PQ/PB-Gel not only can inhibit local tumor recurrence in terms of efficacy, induce systemic immune response and memory T cell formation, but also avoid adverse reactions such as erythrocytopenia after the use of aCD47 in terms of safety. Compared with many preclinical studies on aCD47 that have been reported so far, the use of small molecule inhibitor PQ912 greatly reduces the production cost and can achieve clinical transformation to reduce the economic burden of cancer patients. After loading PQ912 into PB-Gel, it not only has outstanding performance in efficacy and safety, but also has simple preparation method and administration method, and has good practicability. In the future, this simple PQ912 local sustained-release delivery system is expected to become a successor to aCD47, providing a new platform for the postoperative treatment of cancer patients.

## Supplementary Material

Supplementary figures.

## Abbreviations

aCD47: CD47 monoclonal antibody
aPD-1: anti-PD-1 antibodies
BSA: bovine serum albumin
CTLs: cytotoxic T cells
CFSE: carboxyfluorescein succinimidyl amino ester
DCs: dendritic cells
HGB: hemoglobin
ICIs: immune checkpoint inhibitors
IFN-γ: interferon-γ
IL-10: interleukin 10
IL-12p70: interleukin 12p70
IVIS: *in vivo* imaging system
MDSCs: myeloid derived inhibitory cells
MFI: mean fluorescence intensity
MTT: 3-(4,5-Dimethylthiazol-2-yl)-2,5-diphenyl tetrazolium bromide
RBCs: red blood cells (RBCs)
RFP-4T1: 4T1 cells expressing red fluorescent protein
SIRPα: signal-regulating protein α
TAMs: tumor-associated macrophages
Tetra-PEG-SS: tetra-armed PEG succinimidyl succinate
T cell: T lymphocyte
T_CM_ cells: central memory T cells
T_EM_ cells: effector memory T cells
TME: tumor microenvironment
Tregs: regulatory T cells
