# Supplementary material for: Promising alternatives of CD47 monoclonal antibody: an injectable degradable hydrogel loaded with PQ912 for postoperative immunotherapy effectively blocks CD47-SIRPα signal
Source: Theranostics. 2022 May 27;12(10):4581–98. doi: 10.7150/thno.72310 (PMC9254232; doi:10.7150/thno.72310)
Supplement: Supplementary file 1 — Supplementary figures. [file thnov12p4581s1.pdf]

Supporting Information

Promising alternatives of CD47 monoclonal antibody: an injectable degradable hydrogel loaded with PQ912 for postoperative immunotherapy effectively blocks CD47-SIRPα signal

Authors:

Chang Li[1], Yubo Liu[1], Dan Li[1], Qiu Wang[1], Shuang Zhou[1], Haotian Zhang[2], Yongjun Wang[1], Zhonggui He[1], Hongzhuo Liu[1]*, Jin Sun[1]*

Affiliations:

[1]Wuya College of Innovation, Shenyang Pharmaceutical University, Shenyang, 110016, P. R. China.

[2]School of Life Science and Biopharmaceutics, Shenyang Pharmaceutical University, Shenyang, 110016, P. R. China.

*__Corresponding Authors:__

Jin Sun, Ph.D. and Hongzhuo Liu, Ph.D.

Tel/Fax: +86-24-23986321

E-mail address: sunjin@syphu.edu.cn; liuhongzhuo@syphu.edu.cn

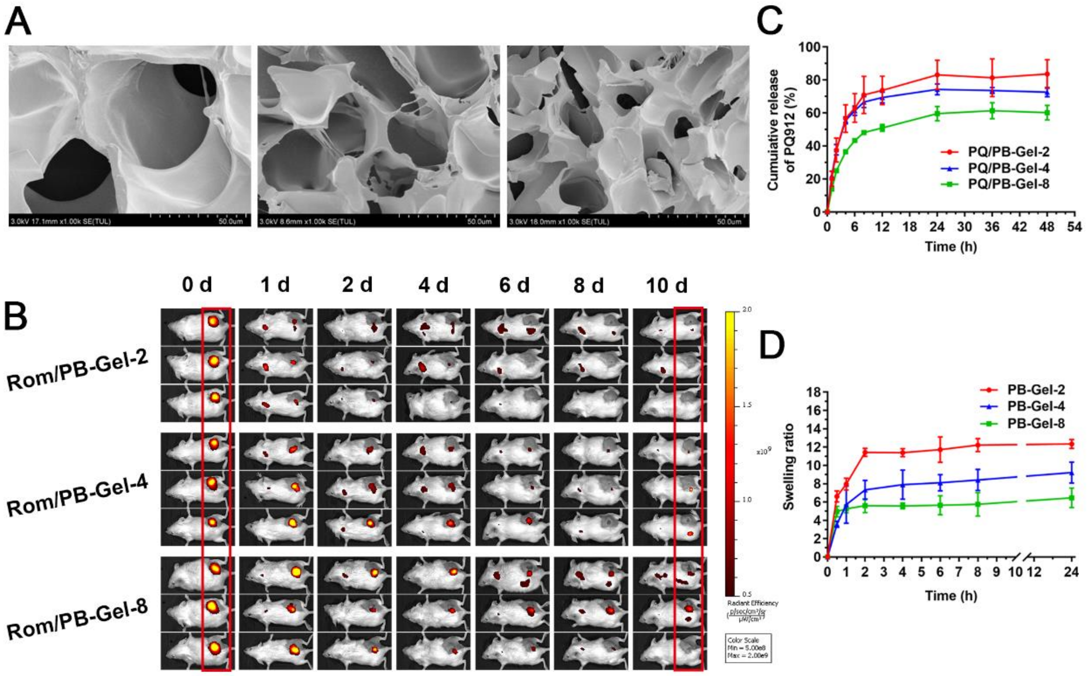

Figure S1. (A) Representative scanning electron microscope images of PB-Gel-2, PB-Gel-4, PB-Gel-8. (B) Three kinds of PB-Gel (Rom/PB-Gel) containing Rom were surgically implanted in non-tumor-bearing BALB/c mice, and the *in vivo* release behavior of Rom in PB-Gel was observed by IVIS spectrophotometric imaging system at a specified time point. (C) Cumulative release behavior curve of PQ912 from PB-Gel. (D) Swelling rate of multiarm polyethylene glycol derivatives. Data shown are means ± SD (n = 3).

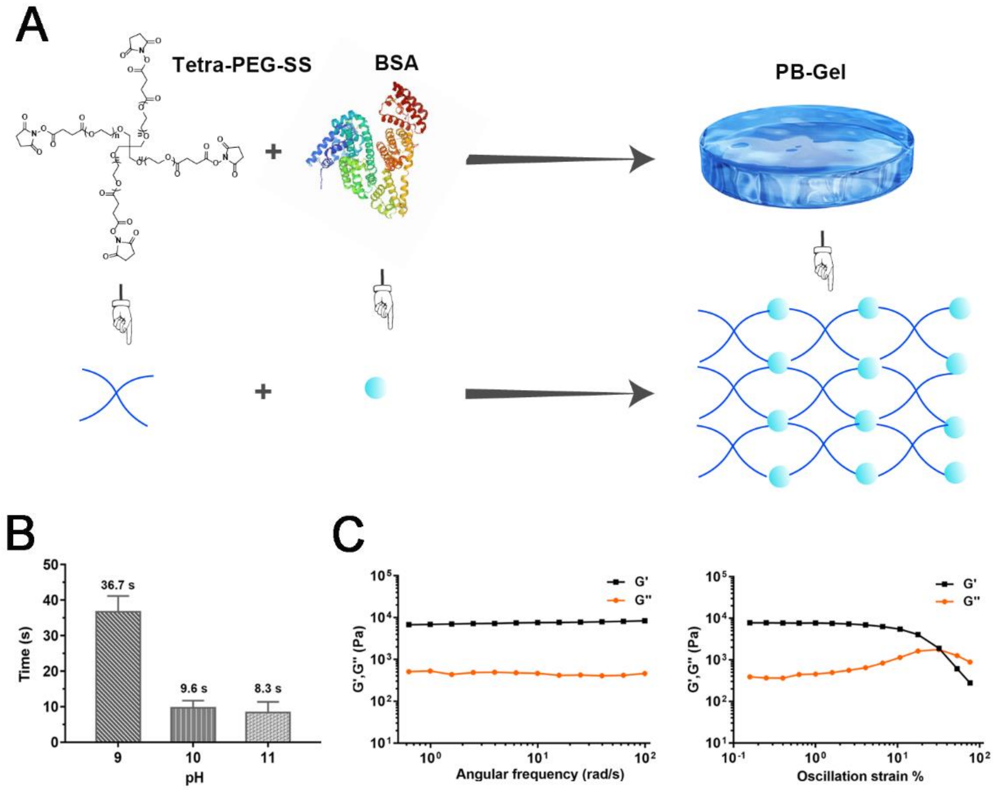

Figure S2. (A) Schematic diagram of hydrogel (PB-Gel) formed by Tetra-PEG-SS and BSA. (B) Effect of pH of BSA solution on the gelation time of PB-Gel. Data shown are means ± SD (n = 3). (C) Dynamic frequency sweep of PB-Gel.

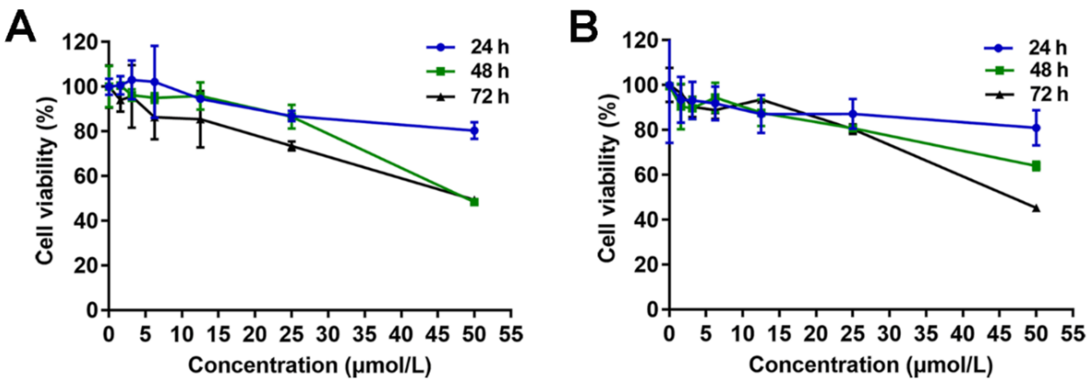

Figure S3. Viability of (A) 4T1 and (B) B16F10 cells treated with different concentrations of PQ912. Data shown are means ± SD (n = 3).

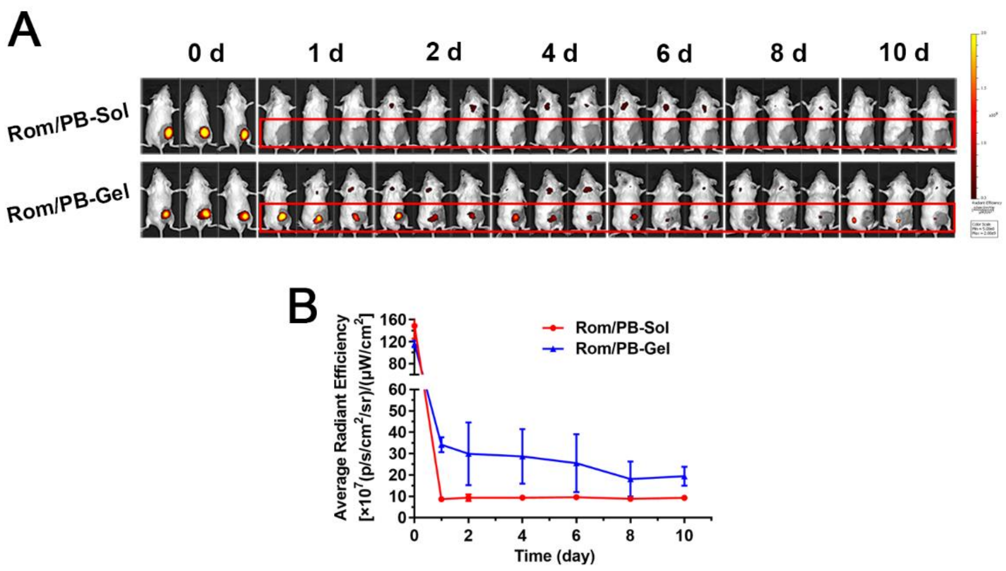

Figure S4. (A) Rom/PB-Sol, Rom/PB-Gel were *in situ* treated surgically into non-tumor-bearing mice respectively and IVIS spectrophotometric imaging system was performed at the specified time points. (B) Quantification of fluorescence IVIS imaging. Data shown are means ± SD (n = 3).

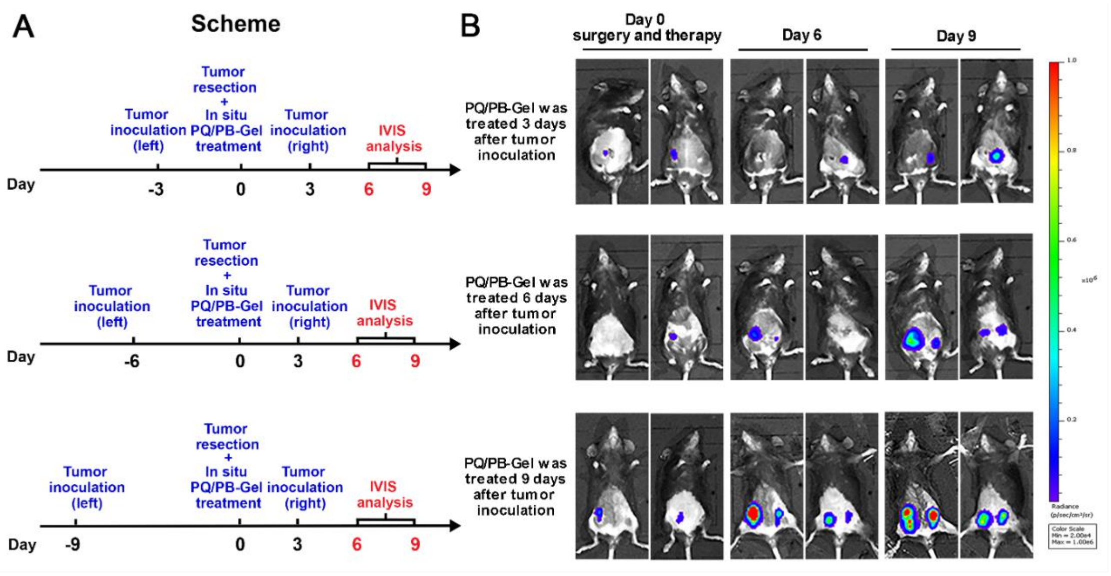

Figure S5. Bioluminescent images of B16F10 melanoma growth after treatment at different times (n = 3).

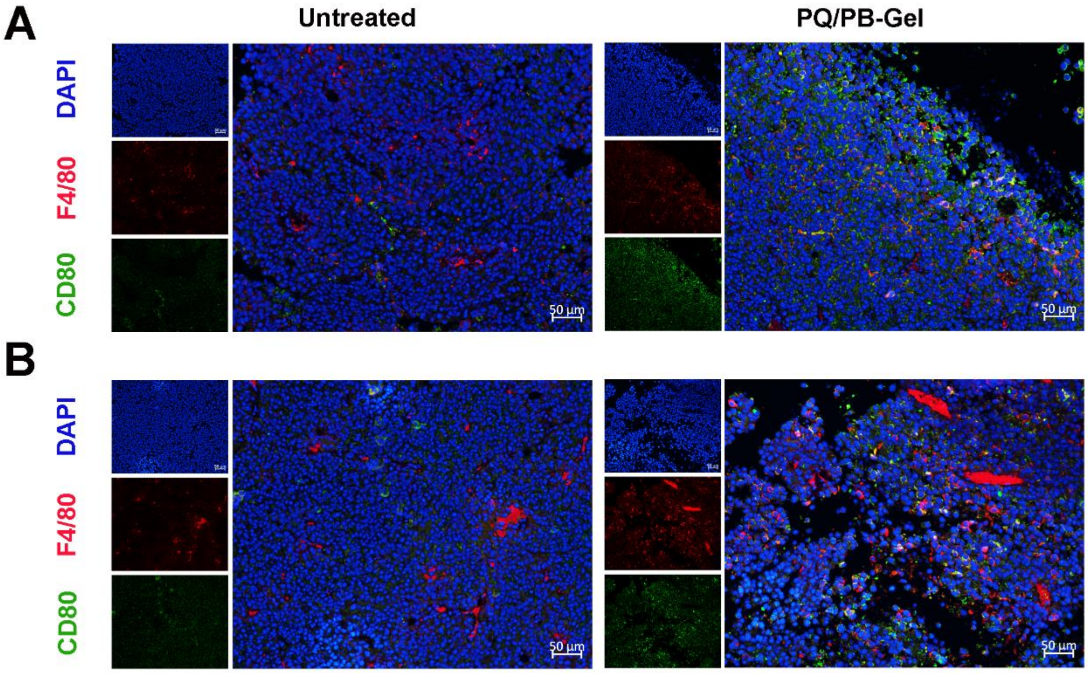

Figure S6. Immunofluorescence of the (A) center and (B) periphery of B16F10 tumor tissue showing $CD80^{+}F4/80^{+}$ cells infiltration. Scale bar, 50 μm.

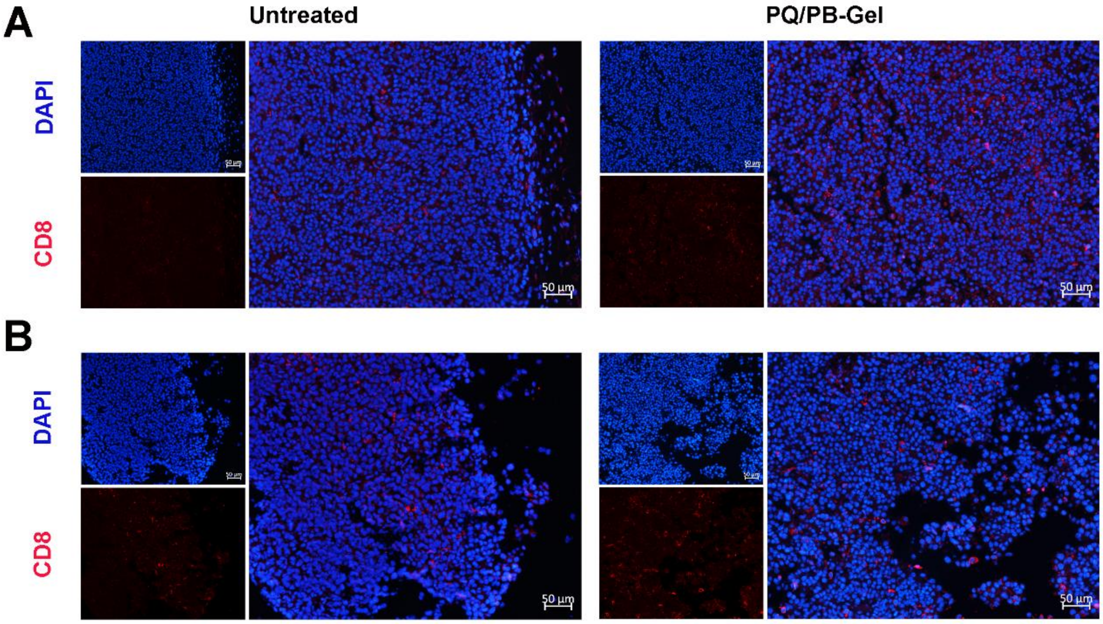

Figure S7. Immunofluorescence of the (A) center and (B) periphery of B16F10 tumor tissue showing CD8$^+$ T cells infiltration. Scale bar, 50 μm.

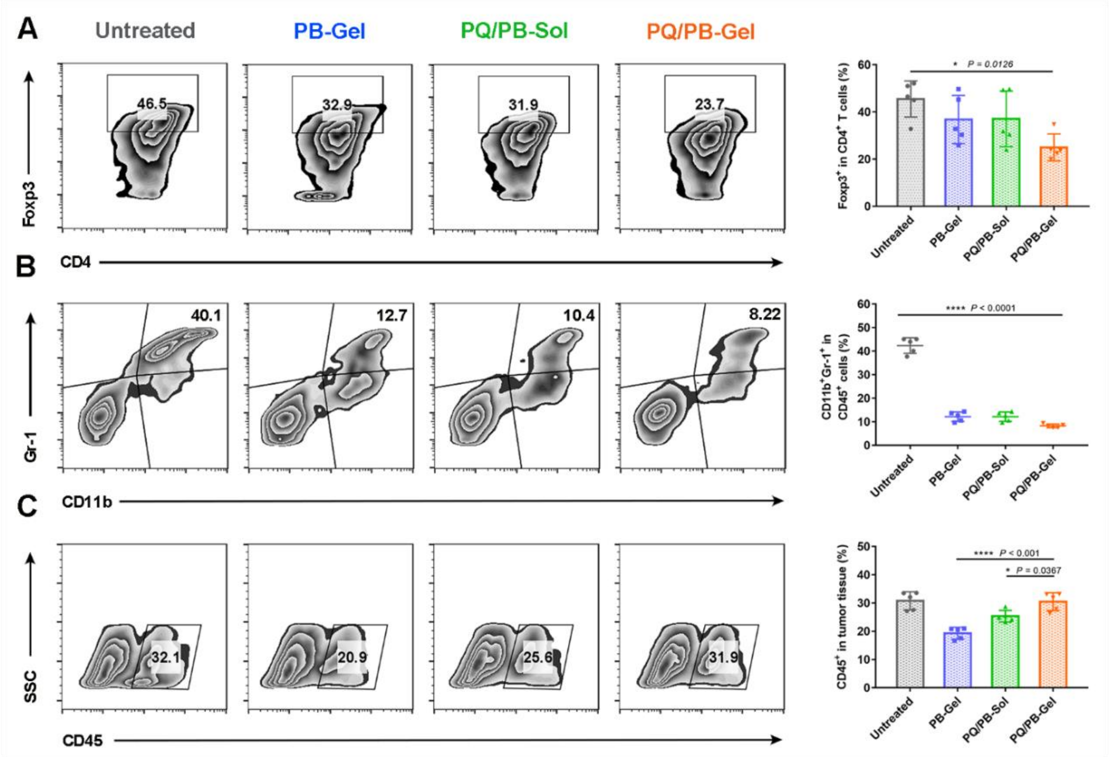

Figure S8. PQ/PB-Gel reversed immunosuppressive TME after B16F10 melanoma surgery. (A) Representative flow cytometric images (left) and relative quantitative images (right) of $CD4^+Foxp3^+$ T cells gating on $CD3^+CD45^+$ cells. Data are presented as means ± SD (n = 5). (B) Representative flow cytometric images (left) and relative quantitative images (right) of MDSCs ($CD11b^+Gr\text{-}1^+$) gating on $CD45^+$ cells. (C) Representative flow cytometry images (left) and relative quantitative images (right) of $CD45^+$ cells. Data are presented as means ± SD (n = 5). Data are presented as means ± SD (n = 5). *$P < 0.05$, **$P < 0.01$, ***$P < 0.001$ and ****$P < 0.0001$ by one-way analysis of variance.

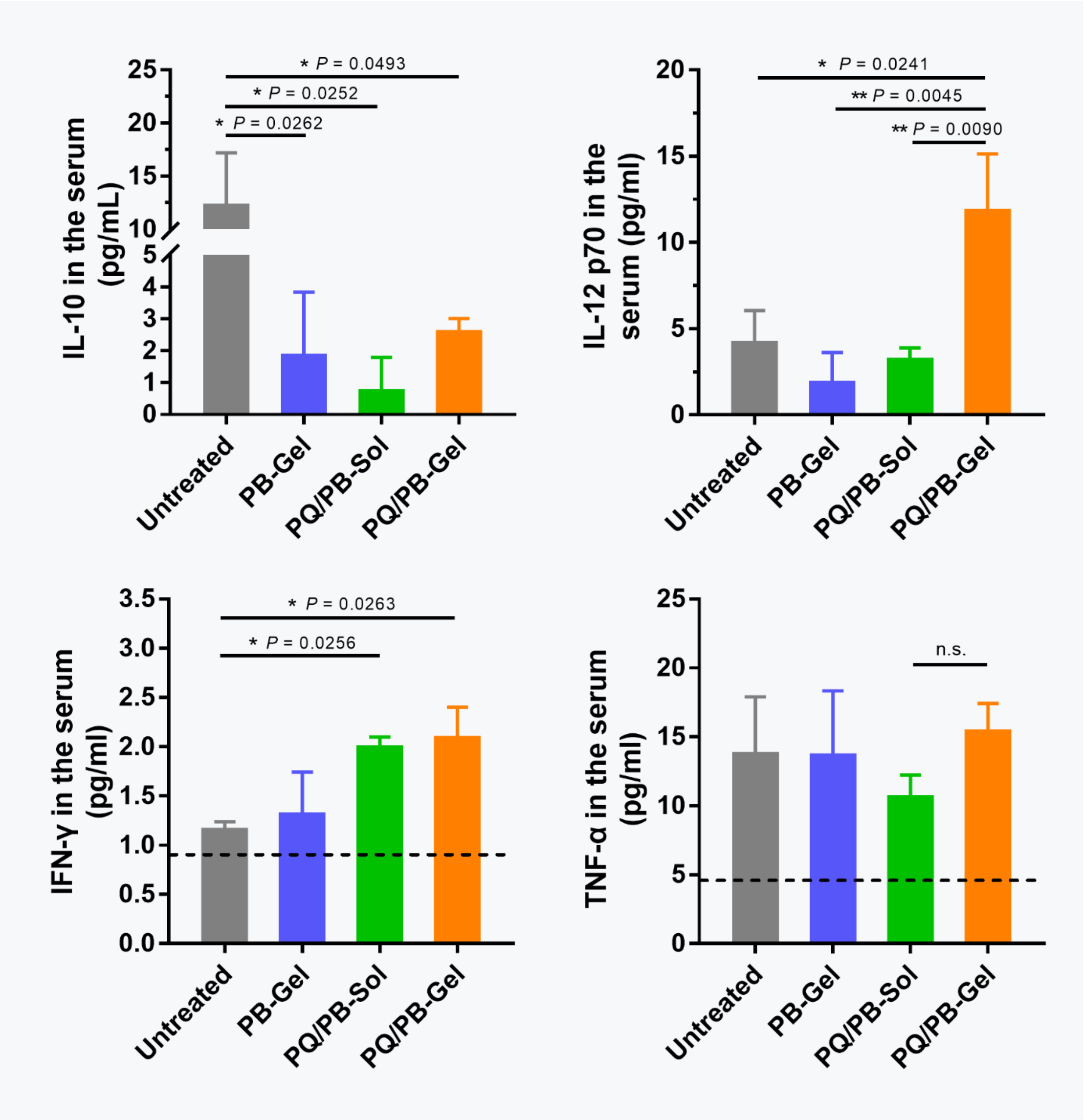

Figure S9. Cytokine levels in the serum from mice isolated 12 days after different treatments. The black dotted line showed the cytokine levels of the mice without tumor. Data are presented as mean ± SD (n = 3). **P* < 0.05, ***P* < 0.01, ****P* < 0.001 and *****P* < 0.0001 by one-way analysis of variance.

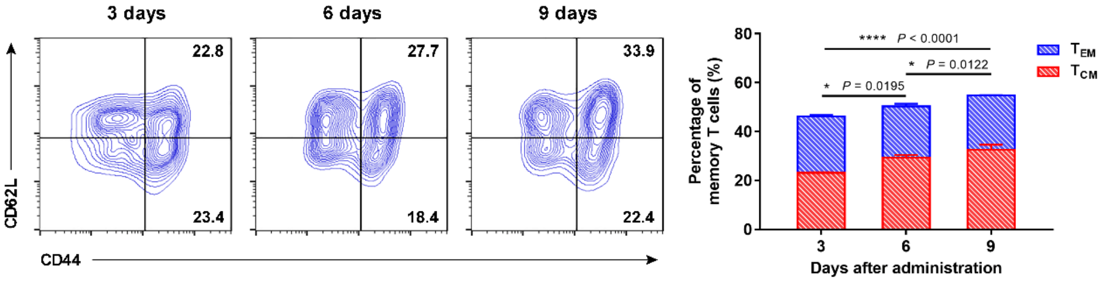

Figure S10. Representative flow cytometric images (left) and relative quantitative images (right) of CD8$^+$ T_{CM} cells (CD44$^+$CD62L$^+$) and T_{EM} cells (CD44$^+$CD62L$^-$) in spleens of mice gating on CD3$^+$ cells in different treatment groups. Data are presented as means ± SD (n = 5). *P < 0.05, **P < 0.01, ***P < 0.001 and ****P < 0.0001 by two-tailed Student's t test.

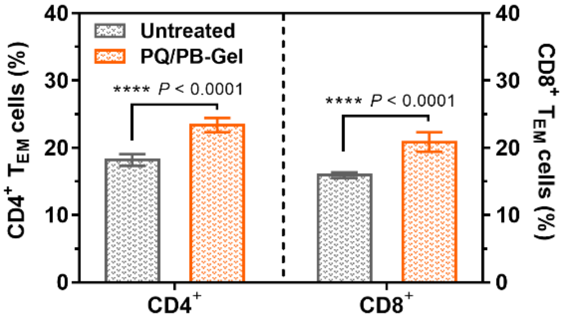

Figure S11. Relative quantitative images of $CD4^+$ and $CD8^+$ T_{EM} cells ($CD44^+CD62L^-$) in spleens of mice of different treatment groups gating on $CD3^+$ cells. Data are presented as means ± SD (n = 5). $^*P < 0.05$, $^{**}P < 0.01$, $^{***}P < 0.001$ and $^{****}P < 0.0001$ by two-tailed Student's t test.

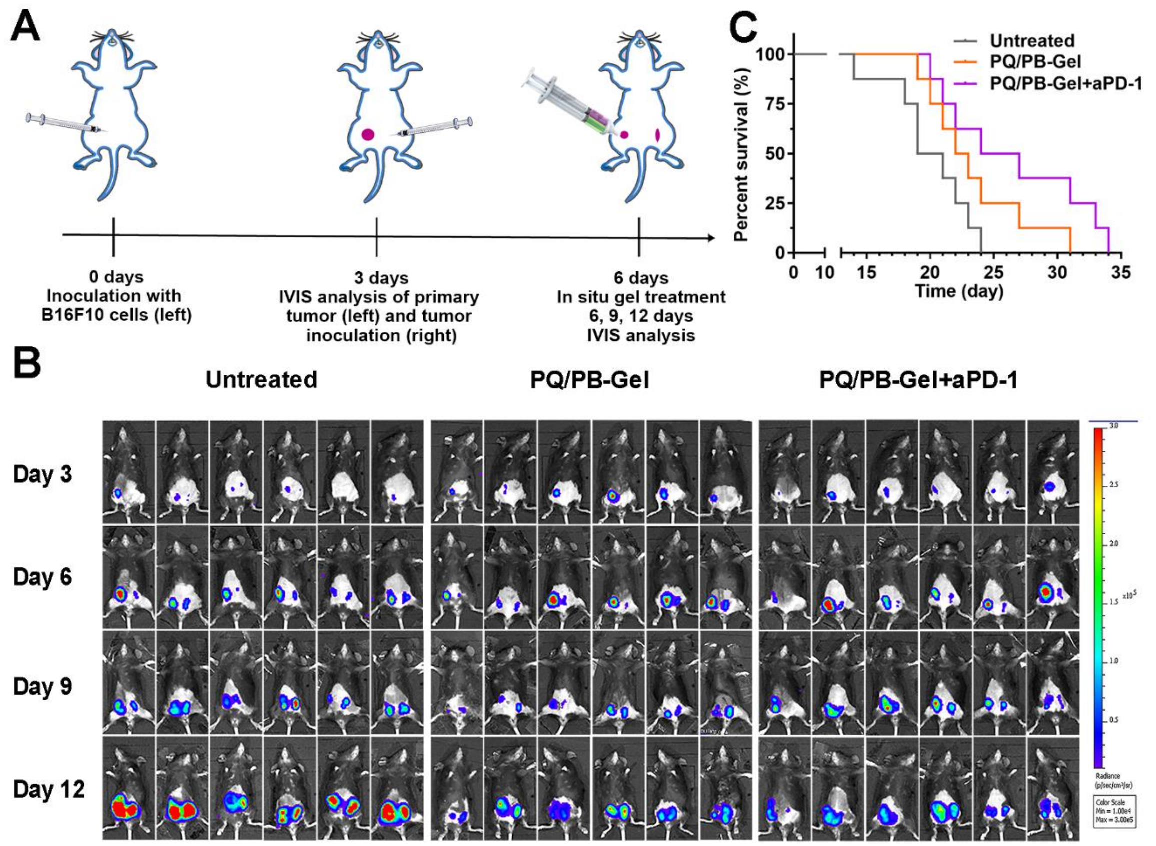

Figure S12. (A) Diagram of treatment schedule. (B) Bioluminescence image of proximal B16F10 *in situ* unresected tumor treated with different treatments in mice (n = 6). (C) Survival of proximal B16F10 *in situ* unresected tumors treated with different treatments (n = 8).

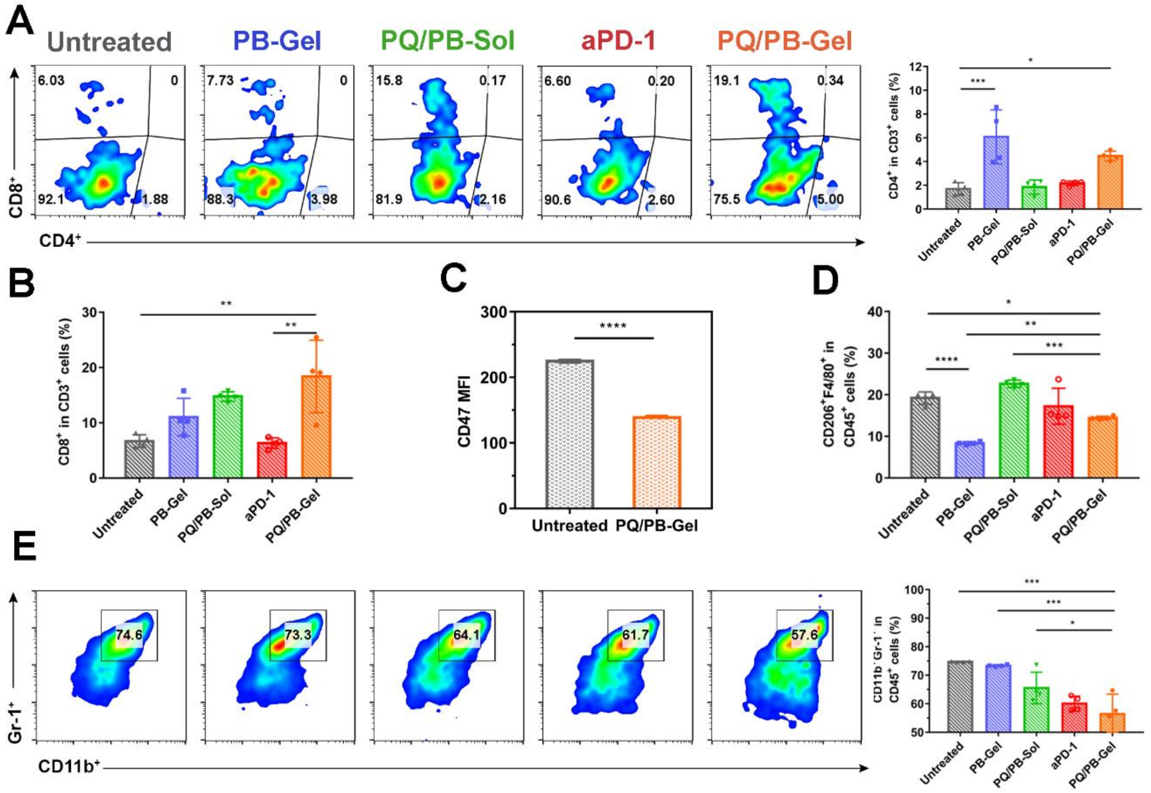

Figure S13. PQ/PB-Gel for triggering antitumor innate and adaptive immune response after 4T1 tumor surgery. (A) Representative flow cytometric analysis of T cells infiltration within the tumor gating on $CD3^+$ cells (left) and relative quantitative images (right) of $CD4^+$ T cells gating on $CD3^+CD45^+$ cells. Data are presented as means ± SD (n = 4) (B) The quantification results of $CD8^+$ cells gating on $CD3^+CD45^+$ cells in different groups. Data are presented as means ± SD (n = 4) (C) Mean fluorescence intensity (MFI) of CD47. Data are presented as means ± SD (n = 5). (D) The relative quantitative images of M2-like TAMs ($CD206^+F4/80^+$) gating on $CD11b^+CD45^+$ cells. Data are presented as means ± SD (n = 4). (E) Representative flow cytometric images (left) and relative quantitative images (right) of MDSCs ($CD11b^+Gr\text{-}1^+$) gating on $CD45^+$ cell. Data are presented as means ± SD (n = 4). *$P < 0.05$, **$P < 0.01$, ***$P < 0.001$ and ****$P < 0.0001$ by two-tailed Student's t test.

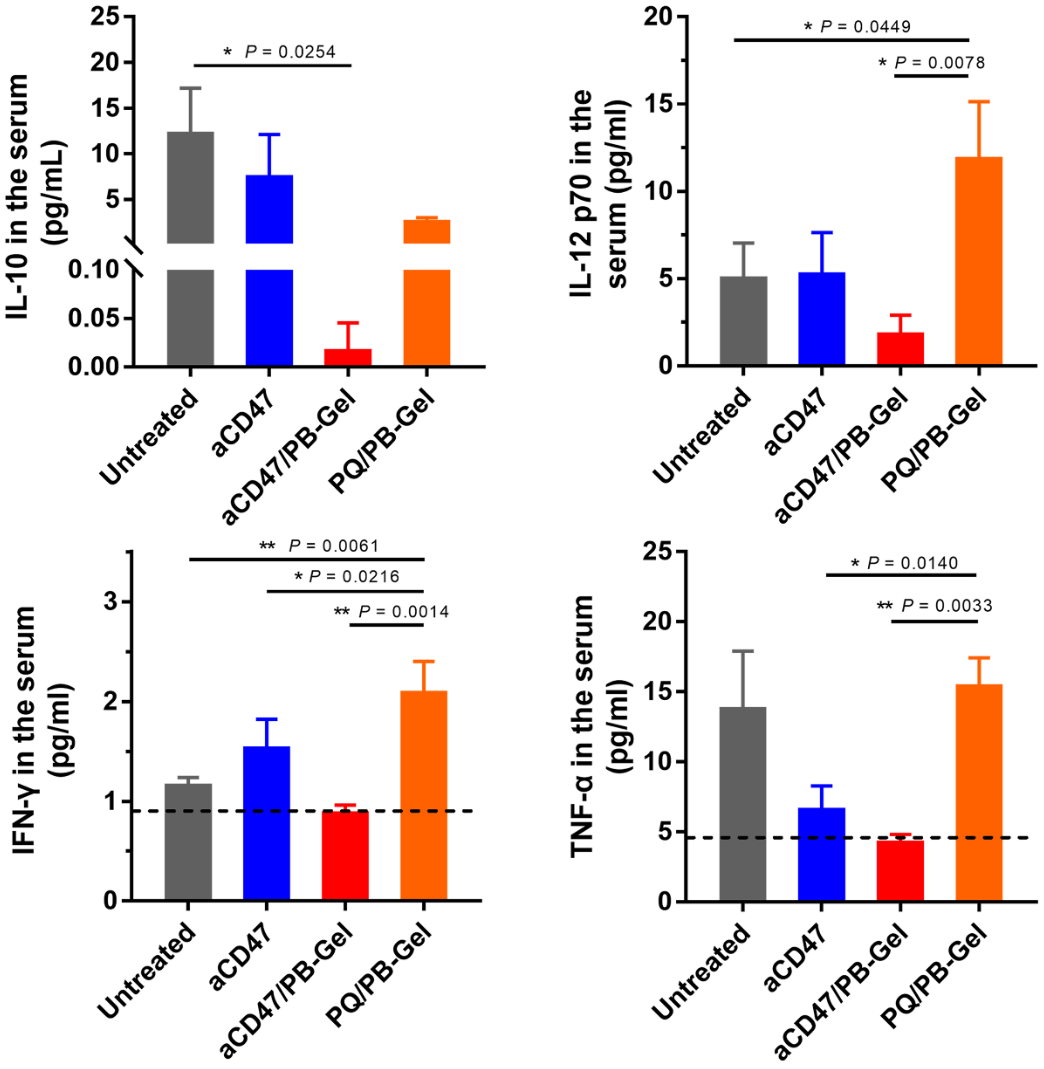

Figure S14. Cytokine levels in the serum from mice isolated 12 days after different treatments. The black dotted line showed the cytokine levels of the mice without tumor. Data are presented as means ± SD (n = 3). *P < 0.05, **P < 0.01, ***P < 0.001 and ****P < 0.0001 by one-way analysis of variance.
